# Materials Research With Neutrons at NIST

**DOI:** 10.6028/jres.106.008

**Published:** 2001-02-01

**Authors:** R. L. Cappelletti, C. J. Glinka, S. Krueger, R. A. Lindstrom, J. W. Lynn, H. J. Prask, E. Prince, J. J. Rush, J. M. Rowe, S. K. Satija, B. H. Toby, A. Tsai, T. J. Udovic

**Affiliations:** National Institute of Standards and Technology, Gaithersburg, MD 20899-8562

**Keywords:** activation-analysis, bio-membrane, condensed-matter, diffraction, neutron, materials, polymer, reflectivity, residual-stress, scattering, vibration

## Abstract

The NIST Materials Science and Engineering Laboratory works with industry, standards bodies, universities, and other government laboratories to improve the nation’s measurements and standards infrastructure for materials. An increasingly important component of this effort is carried out at the NIST Center for Neutron Research (NCNR), at present the most productive center of its kind in the United States. This article gives a brief historical account of the growth and activities of the Center with examples of its work in major materials research areas and describes the key role the Center can expect to play in future developments.

## 1. Introduction

Materials research in the United States is an enterprise linked closely to present and emerging technologies in broad sectors of the economy, including the communications, transportation, aerospace, healthcare, and entertainment industries. NIST has a mission to provide research and standards services matched to the size and importance of these areas in the commerce of the United States. Over the last 50 years the materials research enterprise in the United States has become more coherent, with broadening overlap among science, engineering, and market development activities. At the same time, an increasing role has been played by basic research in physics, chemistry, and biology in the development of new materials and of new uses for established materials. A recent development is the growth of powerful computational facilities (themselves the product of electronics materials efforts) applied to materials science, with an emerging ability to relate microscopic and macroscopic properties to atomic behavior. These developments have increased the need for better tools for the study of atomic and molecular structure and dynamics, a need that is being met by greatly improved scattering probes, including a new generation of synchrotron radiation sources, improved electron-based probes, and improved neutron scattering capabilities.

The 20 MW NIST Research Reactor provides a source for the last of these techniques. Neutrons generated by nuclear fission within the fuel have very high kinetic energies, and are slowed to energies characteristic of room temperature by the heavy water surrounding the fuel. Some are slowed even further to energies characteristic of 30 K by a volume of liquid hydrogen kept near the fuel. The neutrons produced are called thermal and cold neutrons respectively, and they are the ones used in the measurement facilities described here.

In addition to scattering, which is the main technique used at the NIST Center for Neutron Research, neutrons can be used to create new stable or radioactive nuclei by absorption. This property is the basis of Neutron Activation Analysis (NAA), a technique used in Analytical Chemistry for identification and quantitative analysis of isotopic species. Other measurements at the NCNR involve the properties of the neutron itself, such as its lifetime, important to basic physical theories. In this article we will focus on the applications of neutrons to study materials.

### 1.1 What is Measured in Neutron Scattering

Simply, what is actually measured is the number of selected neutrons scattered per unit time from a sample into a detector placed at a given angle with respect to the prepared beam incident on the material. The power of this simple concept becomes apparent when we consider what information flows from such a measurement.

Of the various probes of behavior of condensed matter at the atomic level, uncharged, slowly moving, 1.0087 u, intrinsic spin 1/2, thermal or cold neutrons are very useful, having some unique advantages. For one, they can be well matched in energy and length scales to atomic and molecular processes in matter. For another they are usually weakly coupled probes, penetrating deeply and causing little disturbance or damage to the materials under investigation. A third point is that they are particularly sensitive to hydrogen, which makes them a key probe of the structure and dynamics of organic matter, and therefore of great importance in studying polymers and biological matter. Even more important for many measurements is the fact that hydrogen and its isotope deuterium scatter very differently,^1^ so that full or partial substitution of deuterium for hydrogen allows accentuation of selected parts of molecular systems.

A neutron has wave as well as particle properties. Hence, neutrons of a particular wavelength λ can be selected by means of diffraction at some angle from a “monochromating” crystal, with appropriate filtering of higher diffraction orders, in much the same way as x rays. A beam prepared in this way will have neutrons of nearly uniform wavelength moving in a reasonably well-defined direction, and can be characterized by a momentum ***p***_0_ associated with the wavelength by the de Broglie relation, ***p***_0_ = ℏ***k***_0_, where the modulus of the wave vector ***k***_0_ is 2π/λ_0_. Such a beam, either chopped into pulses or shining continuously, is directed towards a sample that scatters it in some manner, depending on the structure, dynamics, isotopic, and magnetic properties of the material involved. The wavelength of the scattered neutron can also be selected either by a monochromating crystal or by measuring the time-of-flight of a pulse traveling between the sample and the detector placed at a known distance from the sample and angle from the incident beam. One can thus measure the final state momentum ***p***_1_ = ℏ***k***_1_. The momentum transferred to the sample, is called ℏ***Q*** and by the law of conservation of momentum is given by ℏ***Q*** = ℏ***k***_0_−ℏ***k***_1_. Similarly, the energy transferred to the sample in the scattering is given by *E* = ℏ_2_***k***_0_^2^/2*m*−ℏ_2_*k*_1_^2^/2*m*, where m is the mass of the neutron. By measuring these two quantities one can learn a great deal about the structure and dynamics of the material under investigation, as will be described below. Another quantity of interest is the spin state of the neutron that is associated with its intrinsic magnetic moment. Polarized beams (i.e., beams with a well-defined spin orientation) can be prepared, and this orientation can be changed in the encounter with the sample and measured in turn. This property is particularly useful in probing magnetic matter. The selection of incident and final wavelengths and spin states, and hence momentum transfers, energy transfers, and spin angular momentum transfers to the sample for particular measurement purposes is what distinguishes the various neutron scattering instruments at NCNR. Many of these instruments have been discussed in considerable detail in a Special Issue of this Journal, Vol. 98, No. 1, 1993 to which the interested reader is referred for details of measurement technique.

Below we offer a brief history of the neutron source and instrumentation at NIST followed by examples of the kinds of materials research that are current at the Center.

## 2. A Brief History: The First 30 Years

**Fig. 1:**
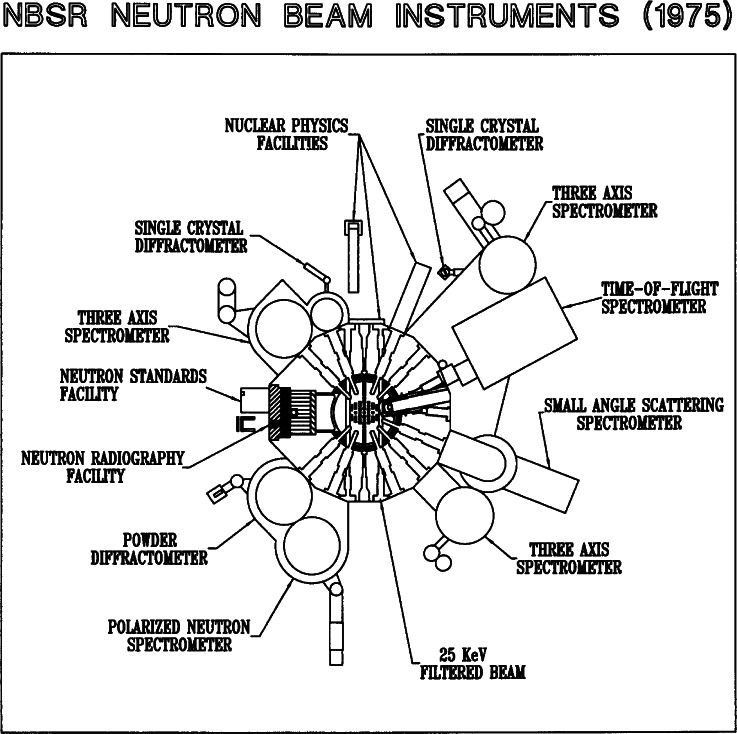
Layout of NBS reactor beams and instruments ca. 1975.

The National Bureau of Standards research reactor (NBSR), which initiated neutron research at 10 MW power in 1969, was engaged from the outset in studies of materials. The genesis of the project a decade before was the recognition that a reactor at NBS would serve the standards and measurements missions of the Bureau, and would act as a regional and national resource to serve other U.S. government agencies, along with universities and industries. The missions and measurement needs of this multidisciplinary community encompassed, even in those years, a varied range of interests in materials, chemical analysis, radiation standards, and other areas. To meet these needs the NBS physicists who designed the reactor, Robert S. Carter, Carl O. Muehlhause, and Harry Landon, integrated three far-sighted features into their plan. The first was a “split-core,” with uranium fuel placed above and below the mid-plane in the heavy-water moderator tank leaving a 17 cm gap in which the thermal neutron flux reaches a peak and other radiation emanating from fission processes is reduced. The insertion of nine large (13 cm to16 cm) radial beam tubes into this gap allowed high intensity beams (with low “background” from unwanted fast neutrons and gamma rays) to be extracted for thermal neutron scattering research. Second, a large volume in the core provided very flexible capabilities for thermal neutron irradiation, and third, a large opening was left in the reactor tank for placement of a “cold source,” which would later become the basis of the Cold Neutron Research Facility (CNRF). This cold neutron capability has made NIST the nation’s leading center for neutron research on materials at the opening of the 21st Century. An early layout of the NBSR and its neutron scattering instruments is shown in Fig. 1.

### 2.1 Early Neutron Scattering Instruments and Activities

The first four instruments for neutron diffraction structure studies were jointly funded by NBS, the Naval Ordnance Laboratory in White Oak Maryland, and the Naval Research Laboratory in Washington D.C. These instruments were driven by the materials research mission activities of the three laboratories. Active programs rapidly developed in studies of new magnetic materials for civilian use as well as for military applications (e.g., sonar transducers), of the structure of glasses, liquids and organic materials, of atomic and molecular scale dynamics, and of phase transitions of orientationally disordered solids and polymers.

The pattern of expanded horizons for research on complex materials continued in the early 1970s when the Army neutron research group of Picatinny Arsenal Laboratory in New Jersey moved its program to the NBSR along with funding to develop two crystal spectrometers for neutron inelastic scattering. These instruments were enormously productive, serving not only Army-driven research on the molecular scale properties of explosive and propellant solids, but enabling NBS to establish a leading role in studies of the diffusion, vibrational dynamics, and phase behavior of hydrogen in metals, and in studies of disordered inorganic materials. Instrumentation improvements also greatly expanded NBSR efforts in magnetism research, including seminal studies of amorphous magnets, with a growing role being played by both local and national universities. Neutron scattering research and partnerships at NIST in the mid 1970s continued to diversify: a strong program was developed to study texture effects in technologically important alloys; the world’s first measurements were made of bulk residual stress in metals and composites; one of the world’s first programs was developed (with the National Institutes of Health, NIH) to study the structure of proteins by neutron diffraction; and one of the earliest studies in the United States of polymer structure by small angle neutron scattering (SANS) was completed. By 1976 collaborators in neutron scattering research at the NBSR included researchers from the Metallurgy, Polymers, Physical Chemistry, and Inorganic Materials Divisions at NBS and from over 20 outside organizations and universities. Throughout the mid-1970s the Reactor Radiation Division also developed an active program in neutron radiography of materials and structures that became one of the major efforts of the NBS non-destructive evaluation program.

During the first decade of reactor operation there were also other strong and growing NBS mission activities. The Bureau placed large groups at the reactor engaging in neutron physics, developing dosimetry standards, and in performing neutron activation analysis (part of the NBS Inorganic Analytical Research Division). The latter group developed a broad-based program in trace analysis methods, with applications ranging from environmental and health research to characterization of standard reference materials. NBS trace analysis facilities were also the basis of long-term efforts at the reactor by the Food and Drug Administration, the FBI, the Geological Survey, and the Smithsonian Institution.

### 2.2 Increased Reactor Power and the Initial Cold Source

Due to the success and growth at the NBSR in materials research and other areas, funding was approved in the late 1970s to double the power of the NBS reactor to 20 MW. Further, at the end of the decade the NBS director chose the development of a SANS facility for the study of macromolecular and microstructure with cold neutrons, as one of the first efforts under the newly created “competence” fund approved by the Department of Commerce (DOC) and Congress. These two developments put the NBS reactor and its research and measurement programs on a different level, and within a few years helped launch a major NBS initiative to create the nation’s first internationally competitive facility for cold neutron research on materials at the NBSR.

During the late 1970s and early 1980s materials research at the NBSR continued to expand both in new applications and in participation within the NBS and nationally. Stimulated by such developments as the SANS diffractometer and a new filter analyzer spectrometer, major new materials research efforts were developed during these years. They included research on the structures of polymers and complex fluids, the dynamics of molecules on the surface of catalysts, and the microstructure of ceramics. This same period saw the success of the Center for Analytical Chemistry at NBS in developing the best capability in the world for using neutron beam methods (neutron depth profiling and prompt-gamma activation analysis) for *in situ* compositional analysis of chemicals and materials.

### 2.3 The Cold Neutron Guide Hall and Modern Developments

**Fig. 2:**
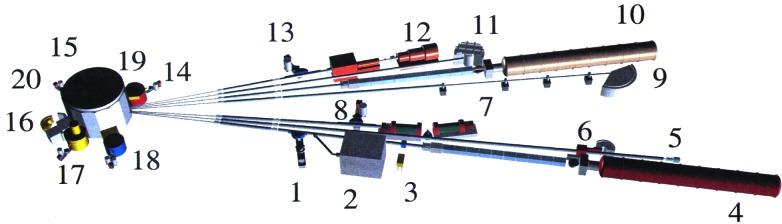
Layout of reactor, guide tubes and instruments used in materials research at the NIST Center for Neutron Research as of 2000. For a detailed description of many of these instruments and their operating principles see the Special Issue of this Journal Vol. **98**, No. 1, 1993. Information about the more recent instruments can be found at http://www.ncnr.nist.gov. (1) NG-7, horizontal sample reflectometer, (2) NG-7, neutron interferometry and optics station, (3) NG-7, prompt gamma activation analysis, (4) NG-7, 30 m small angle neutron scattering (SANS), (5) NG-6, neutron physics station, (6) NG-6, Fermi chopper time-of-flight (TOF) spectrometer, (7) NG-5, spin echo spectrometer, (8) NG-5, spin polarized inelastic scattering spectrometer, (9) NG-4, disk chopper TOF spectrometer, (10) NG-3, 30 m SANS, (11) NG-2, backscattering spectrometer, (12) NG-1, 8 m SANS, (13) NG-1, vertical sample reflectometer, (14) BT-8, residual stress diffractometer, (15) BT-9, triple axis spectrometer, (16) BT-1, powder diffractometer, (17) BT-2, triple axis spectrometer, (18) BT-4, filter analyzer spectrometer, (19) NG-0, cold neutron depth profiling facility, (20) thermal column.

In 1985 and 1987 NBS was successful in budget initiatives first to develop a large cold neutron source and then to build a cold neutron guide hall and guide tube network along with 15 new instruments that provided the United States for the first time with world-class capabilities in cold-neutron research on materials. The 1990s saw major improvements in thermal neutron instrumentation, including a state-of-the-art high-resolution powder diffractometer and a residual stress diffractometer. These improvements, combined with the opening in 1990 of the cold neutron research facility, with instrument developments continuing through the 1990s, made the NBS reactor facility (now called the NIST Center for Neutron Research, NCNR) the most heavily utilized neutron center in the United States. In fact, by the late 1990s, largely through its carefully developed users’ program, the NCNR was host to over half the neutron researchers in the country, with 1600 research participants a year from 250 universities, industries, and laboratories from around the nation and the world. The impact of neutron measurements on NIST mission activities also grew correspondingly to participation by seventeen divisions and offices in 1999. The need for the new neutron measurement probes provided at NIST is attested to by the fact that participants grew by almost a factor of four in the last decade of the century. A layout of the instrumentation at NCNR at century’s end is shown in Fig. 2.

### 2.4 Research Activities in the Last Decade of the 20th Century

The role of NCNR and other NBS scientists in key neutron scattering studies of new materials accelerated in the late 1980s and 1990s, in part due to new instrumentation and new research leaders who came to NIST during the creation of an expanded center. For example, some of the most important U.S. studies of the structure and physics of high *T*_c_ superconductors were carried out with the new powder diffraction and crystal spectrometry instruments. NIST scientists and their collaborators also did the earliest neutron vibrational spectroscopy, and the leading work elucidating the molecular dynamics and its relation to structure in buckminsterfullerene and its related compounds. Work continues in this area on the related buckeytube materials. The 1990s also saw a significant expansion of advanced research in polymers, colloids, and other complex fluids at NIST, in part due to the development of world-leading neutron reflectometer facilities in the cold-neutron guide hall to join the small angle scattering capabilities at the NCNR. In recent years researchers working at the NCNR have received six major prizes in macromolecular and surface science in testimony to these developments in instrumentation and scientific programs.

**Fig. 3:**
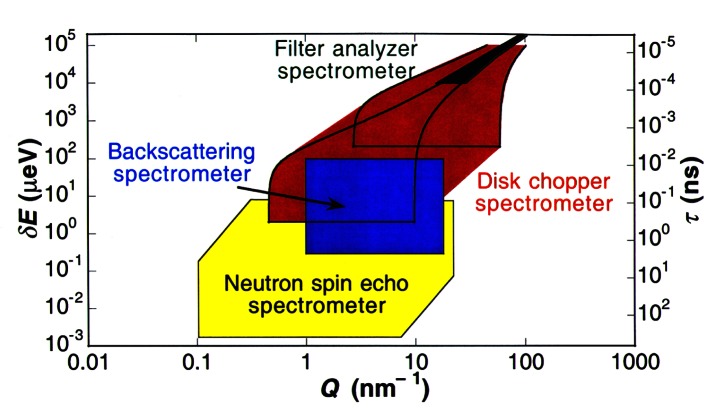
Regions of the energy transfer and time scale, and momentum transfer and length scale variables that can be accessed by various spectrometers at the NCNR.

The neutron measurement and science capabilities at the NCNR have since the late 1980s attracted a number of instrumentation partnerships in the materials research area, including alliances with NSF (the Center for High Resolution Neutron Scattering, CHRNS), Exxon (now ExxonMobil), IBM, University of Minnesota, University of Maryland, University of Pennsylvania, and John Hopkins University. In line with the mission and culture of NIST, NCNR scientists, and their partners have also played a special role in developing new approaches and instrumentation to advance measurement technology in the neutron field for applications in materials science, physics, chemistry, and biology. NIST innovations in the last ten to fifteen years include: development of more efficient next generation hydrogen cold neutron sources; new optical and beam focusing devices to allow much higher intensity for measurements by cold neutron SANS and spectroscopy; development of neutron reflectometry devices to allow measurement of biological, magnetic, and semiconductor interface structures with unparalleled sensitivity and resolution; use of phase-space transform concepts to provide the world’s best measurement capability for neutron backscattering spectroscopy; and development of a next-generation neutron vibrational spectrometer with one hundred times previous sensitivity for studies of materials dynamics. The result of these and many other improvements at the NCNR have greatly enhanced U.S. neutron measurement capabilities, including a three order of magnitude increase in the range of time and energy accessible for studies of materials and molecular dynamics and two orders of magnitude in sensitivity for small angle scattering studies. The ranges in *Q* and *E* (discussed in Sec. 1.1) as well as overlaps of the various instruments available at century’s end at the NCNR for dynamics studies are shown in Fig. 3.

### 2.5 Prospects for the First Quarter of the 21st Century

As NIST enters a new century, we are using the advances in neutron source development and instrumentation described above to open up a new U.S. capability in high-resolution cold neutron spectroscopy of materials, molecules, and macromolecules. Improvements in cold sources, optics, and crystal spectrometers are under development that will extend *Q* and *E* measurement regimes to allow advances in condensed matter physics, macromolecular science, and in a number of other fields. Finally, the process is well underway to extend the operating license of the NIST reactor to continue to serve U.S. science and technology through the first quarter century of the new millennium.

## 3. Biological and Biomimetic Materials

### 3.1 Brief Overview and Early History

Since neutrons are sensitive to the positions of the light elements such as H, C, N, and O, which are of central importance to all biological systems, neutron scattering can provide unique information on the structure and function of biological macromolecules. Particularly powerful is the contrast variation technique, in which the isotopic substitution of D for H is routinely used to change the scattering from a macro-molecule without affecting its biochemistry. This substitution can be as simple as using D_2_O instead of H_2_O in the solvent or as complex as specifically deuterating individual amino acids or entire subunits in a protein complex. The NCNR has been involved in the study of biological materials using neutrons for the last 20 years. In the past decade, it has become a major U.S. center for the structural study of macromolecules and biomimetic materials in solution. With the commissioning of three state-of-the-art inelastic scattering spectrometers in the past year, the NCNR stands to be a major force in the area of macromolecular dynamics as well.

In the early 1980s some of the first neutron work in structural biology was being performed at Brookhaven National Laboratory in Upton, NY and at what was then the NBS reactor. An instrument [1] dedicated to neutron diffraction from protein crystals was built at the NBS reactor, making it possible to locate hydrogen atoms in protein crystals. This led to important studies with scientists at the NIH [2,3] in which x-ray and neutron diffraction data were refined simultaneously to obtain complete maps of the protein structures. Since the location of each hydrogen atom is known, studies of the active site of ribonuclease A (RNase A) [4] (and of hydrogen exchange in RNase A [5] were possible. Thus, structural biology at NIST was done first at the NBS reactor. A productive program in neutron protein crystallography continued until the reactor was shutdown for the installation of the guide hall and original cold source.

### 3.2 SANS and the Contrast Variation Technique

Small-angle neutron scattering^2^ (SANS) has been used to study the solution structure of biological macromolecules for over two decades. Since SANS measurements are performed in solution, the technique can provide unique structural information under conditions that more closely mimic the molecule’s natural environment. With the opening of the guide hall and the availability of a 30 m SANS instrument on a cold neutron guide, studies of biological macromolecules in solution became feasible at the NCNR. At the same time, recent advances in biochemistry, crystallography and structural nuclear magnetic resonance (NMR) were making it possible to prepare greater quantities of deuterium-labeled proteins and to determine an ever-increasing number of high-resolution structures. Thus, SANS came into wider use as a complementary tool for comparing structures in the crystal and solution phases and for elucidating the unresolved regions in a crystal structure.

The contrast variation [6] technique is particularly powerful for studying structural changes in proteins upon binding of nucleotides, lipids, peptides or co-factors. Since the scattering from one component can be separated from that of the other by substituting D for H in the molecule and/or the solvent, the conformations of the macromolecule bound in the complex and free in solution can be compared. This could lead to a better understanding of the biomolecular function of the molecule in the complex. Scientists from Los Alamos National Laboratory regularly come to the NCNR to use neutron scattering and contrast variation to study the mechanism of action of protein complexes important for muscle regulation [7–11]. This same group has also studied the Ku protein/DNA complex [12]. A group of scientists at Rice University in Houston, TX have made progress studying the location of large peptide complexes in lipid bilayers [13] using the 30 m SANS instruments at the NCNR. In addition, there are several ongoing collaborations with NCNR scientists and groups at the Center for Advanced Research in Biotechnology in Rockville, MD. Two of these current SANS projects are described below.

### 3.3 Molecular Chaperonins

Molecular chaperonins, which include GroEL, are essential cellular components. Chaperonins not only protect proteins against stress conditions that cause denaturation but also bind to unfolded proteins to help them fold into their active conformations. GroEL consists of 14 subunits of relative molecular mass *M*_r_ = 57 400 each, for a total *M*_r_ greater than 800 000. The crystal structure of GroEL at 0.28 nm resolution [14] has shown that seven of the subunits are assembled into a ring that has an outer diameter of 13.7 nm and an inner diameter of 4.5 nm. The other seven subunits form an identical ring and the two rings combine to form a cylindrical molecule with a height of 14.6 nm. Sixteen C-terminal amino acid residues (*M*_r_ = 40 600) are unresolved in the crystal structure, presumably due to their disorder.

The role of molecular chaperones in mediating and controlling intracellular as well as *in vitro* protein folding has broad implications for biotechnology. One of the key issues in establishing a molecular mechanism for GroEL is to describe in structural terms the conformations of polypeptide substrates when bound to GroEL. In an effort to examine the location and the conformational properties of a polypeptide chain substrate in chaperonin complexes, SANS experiments with contrast variation were undertaken at the NCNR by CARB (Center for Advanced Research in Biotechnology) and NCNR scientists, using a 86 % deuterated nonnative polypeptide substrate (dPJ9), and a protiated GroEL single-ring variant (GroEL_K105E_). Analyses of these data were enhanced by two parallel studies. The first was of a mutational variant of GroEL in which the 16 C-terminal residues were absent. The second was a SANS study of double ring GroEL complexes as found in nature [15]. These additional data sets enabled an assignment of the crystallographically disordered C-terminal domain of GroEL, and were also helpful in modeling studies of the solution structure of the single ring chaperonin.

Extensive contrast variation SANS data for the GroEL_K105E_/dPJ9 complexes were obtained in buffers containing (0, 20, 70, 85, and 100) % D_2_O. The data were analyzed in terms of scattering from the complex, as well as from the separate components [16]. The scattered intensities of the three contributions were found using the equation,

$$\begin{array}{c} I(Q)=\Delta {\rho_{\text{EL}}}^{2}I_{\text{EL}}(Q)+\Delta \rho_{\text{EL}}\Delta \rho_{\text{dPJ}9}I_{\text{ELdPJ}9}(Q)+\\ \Delta {\rho_{\text{dPJ}9}}^{2}I_{\text{dPJ}9}(Q), \end{array}$$ (1)

where *I*(*Q*) is the measured scattered intensity at momentum transfer *ℏQ* and the contrast Δ*ρ* = (*ρ*−*ρ*_s_) is the difference between the mean scattering length density^3^ of the molecule *ρ* and that of the solvent *ρ*_s_. Here, *I*_EL_(*Q*) and *I*_dPJ9_(*Q*) refer to the scattering intensities of the GroEL_K105E_ component and the dPJ9 component of the GroEL/dPJ9 complex, respectively. The cross-term *I*_EldPJ9_(*Q*) represents the interference function between the GroEL_K105E_ and dPJ9 components and can be used to estimate the distance between the centers of mass of the two components in the complex. The contrasts Δ*ρ*_EL_ and Δ*ρ*_dPJ9_ between each component and the solvent are known for each of the five contrasts measured. Since there are five equations but only three unknowns, *I*_EL_(*Q*), *I*_ELdPJ9_(*Q*) and *I*_dPJ9_(*Q*), the problem is over-determined and values were obtained at each *Q* value by a least squares fitting method.

**Fig. 4:**
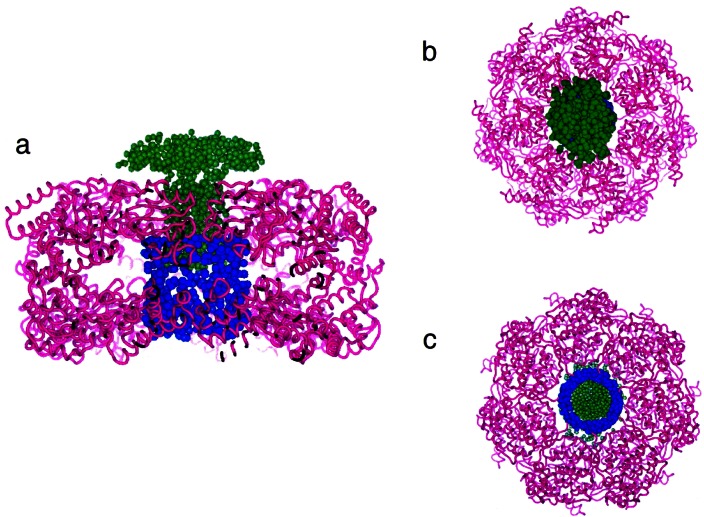
Drawing of the structure of the single ring GroEL_K105E_/dPJ9 complex. The ribbon structure was obtained from the x-ray crystallographic coordinates whereas the structures rendered as balls, the PJ9 (green), and the disordered C-terminal residues of the GroEL (blue), were determined from SANS measurements. (a) Cross-sectional side view, (b) top view, (c) bottom view.

A comparison of the scattered intensity for GroEL_K105E_ measured free in solution with that bound in a complex indicates that little or no change in GroEL conformation occurs upon binding the substrate polypeptide. Monte Carlo molecular modeling approaches indicate that a mushroom-like conformation for dPJ9 yields theoretical scattering data that agrees well with the experimental data, similar to results obtained for rhodanese [15]. In fact, the model fits the data for the GroEL_K105E_/dPJ9 complex at all contrasts, including H_2_O, where the scattering contrast from the dPJ9 is the highest. This indicates that the shape and location of modeled dPJ9 in the complex are reasonable. Pictures of the dPJ9 shape and location in the complex are shown in Fig. 4, along with the most probable location and shape of the crystallographically disordered C-terminal residues.

### 3.4 Conformational Changes in a Gene Regulatory Protein

DNA molecules direct the synthesis of specific RNA and protein molecules, which in turn determine a cell’s chemical and physical properties. Specific regions of the DNA (genes) contain the information, or code, that is required for the synthesis of proteins or enzymes. In the early stages of protein synthesis, genes of DNA are copied into short strands of RNA which retain all of the genetic information of the DNA sequence from which they were copied. These short RNA strands contain enough information for the synthesis of one or, at most, a few proteins. The process by which RNA molecules are synthesized from the coding regions of DNA is known as DNA transcription. The RNA polymerase enzyme, whose function is to make a RNA copy of a DNA sequence, catalyzes the synthesis of these RNA molecules.

In any cell at any given time, some genes are used to make RNA in very large quantities while other genes are not transcribed at all. In other words, a specific set of genes may be turned “on” while others remain “off.” The amount of RNA made from a particular region of DNA is controlled by gene regulatory proteins that bind to specific sites on DNA close to the coding sequences of a gene. One useful model of such a protein is the cyclic AMP receptor protein (CRP) of *E. coli.* The functional unit of CRP is a dimer with a relative molecular weight *M*_r_ = 42 000. Upon binding cyclic adenosine monophosphate (cAMP), CRP undergoes a conformational change that, in turn, promotes binding to specific DNA sequences that code for over twenty enzymes involved in sugar metabolism. The CRP/cAMP complex, upon binding DNA, produces a bend in the DNA that causes it to wrap around the RNA polymerase to promote DNA transcription.

CARB and NCNR scientists are using SANS along with thermodynamics, protein engineering, and biophysical characterization methods in order to better understand the molecular mechanism of cAMP-induced conformational changes in CRP. The crystal structure of the CRP/cAMP, the “ligated” protein, suggests that both “open” and “closed” conformations can exist. But only the closed form is known to bind DNA. The question addressed is: which conformations of the ligated and unligated (i.e., without cAMP) proteins exist in solution? SANS data [17] were obtained for CRP partially ligated with cAMP and unligated protein in D_2_O solvent. The unligated protein SANS data are especially important since a crystal structure is available for the ligated protein [18] but not for the unligated one.

From the SANS data a small decrease in radius of gyration, *R*_g_, from (2.20 ± 0.06) nm to (2.15 ± 0.04) nm, was observed for the ligated protein sample compared to the unligated one. However, observable differences were found in the SANS data at higher scattering angles indicating differences in structures on a smaller length scale.

To determine which conformations of the ligated and unligated protein exist in solution (as distinct from the crystalline case), molecular dynamics simulations [17] were performed in order to obtain energy-minimized structures of both open and closed forms of the ligated protein and of the unligated protein. To provide a starting model for the unligated form in the simulation, the cAMP molecule was removed from the ligated protein crystal structure. SANS intensity profiles were then calculated using a Monte Carlo technique [19] for all models, i.e., using the coordinates of the calculated structures of both open and closed forms of both ligated and unligated proteins, and compared to data.

The minimized structures obtained for the ligated protein are similar for both open and closed forms, and the SANS data agree with both reasonably well. For this reason the state of CRP ligated with cAMP cannot be ascertained from the SANS data alone. However, based on the concentration of CRP relative to the cAMP in the sample, it is likely that this particular partially ligated sample exists as a mixture of both open and closed states. On the other hand, comparisons of the open and closed model SANS profiles to the data indicate that CRP unligated with cAMP clearly exists in the open form in solution and so cannot bind DNA.

**Fig. 5:**
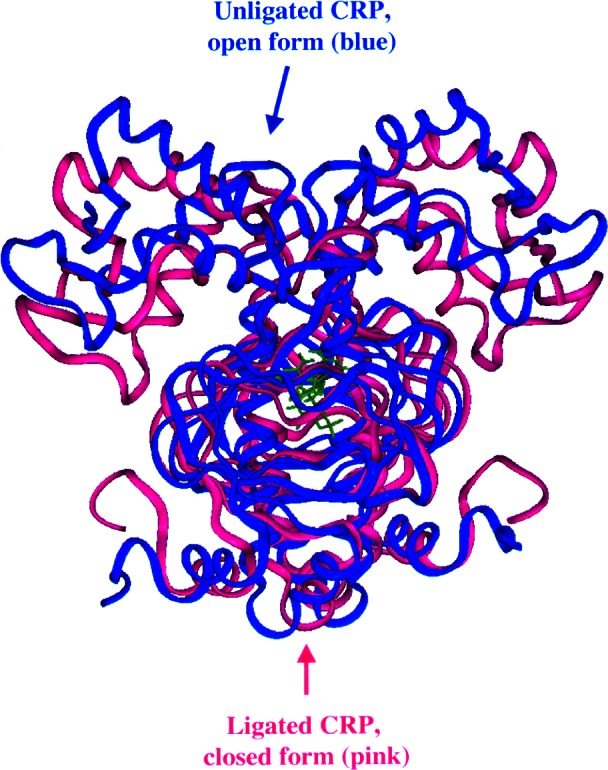
Comparison of two model structures of the cyclic AMP receptor protein (CRP). The pink ribbon represents the closed form, which binds DNA, of CRP ligated with adenosine monophosphate (cAMP), and the blue ribbon represents the open form, which cannot bind DNA, of unligated CRP. The ligated structure (pink) was obtained by performing a molecular dynamics minimization starting from the known x-ray crystallographic structure of the cAMP-ligated form of the protein. The unligated structure (blue) was obtained by removing the cAMP from the x-ray structure before molecular dynamics minimization. SANS curves were calculated for each model and compared to the measured SANS data. This comparison indicates that the unligated protein clearly exists in the open form in solution.

Figure 5 shows a drawing of the structures of the open form of the unligated protein and the closed form of the ligated protein that best fit the SANS data. Current experiments are making use of the contrast variation technique to verify the bending of the DNA in cAMP ligated CRP/DNA complexes [20] and to model changes in the protein structure upon DNA binding [21].

### 3.5 Biomimetic Membranes

A biological membrane that defines its boundaries and regulates its interactions with the environment encloses each cell. The biological membrane consists of assemblies of lipid and protein molecules. The lipid molecules form a continuous double layer, or bilayer, which acts as a barrier to water-soluble molecules and provides the framework for the incorporation of the protein molecules. Specialized proteins embedded in lipid bilayers participate in fusion events between cells (i.e., triggered by viruses), regulate ion transport through pores and channels (i.e., neural activities), engage in enzymatic activity at membrane surfaces, and play a role in biological signaling (i.e., receptor proteins activated by hormones). Cell membranes are sufficiently complicated that they cannot be duplicated in the laboratory for study. Thus, model biological membranes, which are simpler than cell membranes but mimic their structure and function, are used to study these complicated systems. Such model membranes are known as biomimetic materials, which emulate biological function such as molecular recognition, dynamic conformational change and spontaneous self-assembly of complex arrays of molecules.

**Fig. 6:**
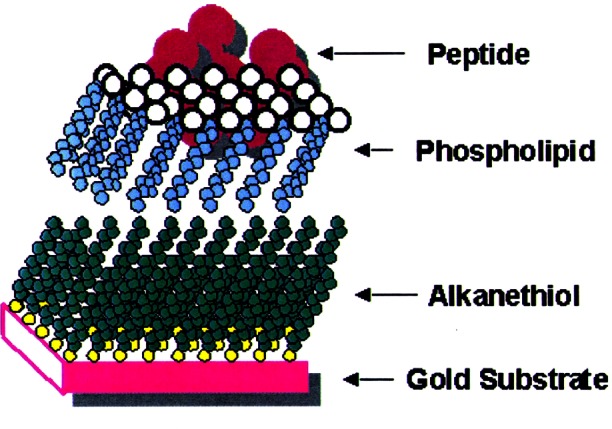
A schematic drawing of an alkanethiol/phospholipid hybrid bilayer membrane (HBM) with an embedded membrane-active protein. For neutron reflectivity experiments, the HBM is formed on a 10 cm diameter silicon wafer, which was coated with ≈5 nm gold on a 1 nm to 2 nm chromium adhesion layer. The HBM is in direct contact with aqueous solution, usually consisting of a mixture of D_2_O and H_2_O.

A biomimetic material that is analogous to the lipid membranes of cells and can support active membrane proteins has been made in NIST’s Biotechnology Division. This hybrid bilayer membrane (HBM) consists of two self-assembling monolayers, one which is non-biological (alkanethiol) and a second which can be found in biological cell membranes (phospholipid), and is formed spontaneously on a planar gold surface. Since the alkanethiol monolayer is strongly bonded to the gold surface, this HBM is more rugged than a conventional supported phospholipid bilayer, which binds only weakly to a silicon or glass surface. In addition to their obvious importance as a tool for understanding and characterizing membrane protein structure and function, the biomimetic characteristics of the HBMs make them commercially significant for a number of applications including biosensors, tissue engineering, and bio-electronics and biocatalysis. The lipid and protein composition of the HBM can be readily engineered to produce structures with novel physical and chemical properties that do not occur in nature. A schematic of the alkanethiol/phospholipid HBM with an embedded membrane-active protein is shown in Fig. 6.

The process of optimizing the biomimetic character of these membrane matrices requires the development of measurement tools for probing the structure and function of these engineered matrices and the cell membrane components incorporated into them. To this end, the neutron reflectivity technique is being developed at the NCNR to assist in the structural characterization of HBMs in contact with aqueous solution. This has required advancements in instrumentation, sample environment, and measurement protocols, which should be applicable to other surface and thin film studies that demand measurements in aqueous solution. The first neutron reflectivity measurements from a HBM system were performed on the BT-7 reflectometer [22], before an improved instrument was reconstructed in the guide hall.

Current neutron reflectivity measurements are being made on the NG-1 reflectometer at the NCNR. A novel sample cell design makes it possible to obtain tenth nanometer-level information about the composition of HBMs along the *z* axis, perpendicular to the plane of the membrane. The most recent measurements have been made on a HBM matrix consisting of an ethylene oxide-containing alkanethiol monolayer (THEO-C18) and a phospholipid (dDMPC) monolayer. The ethylene oxide moiety tethered to the gold surface acts as a hydrophilic “spacer,” and would be expected to be more fluid than the alkanethiol region, thus providing a hospitable environment for the incorporation of water and transmembrane protein segments near the gold surface. Measurements have been made both in the absence and presence of the membrane protein, melittin [23,24], a relatively small peptide toxin that is found in bee venom. Melittin, an important model compound for pore-forming peptides such as antibiotics, is water-soluble and partitions from solution into preformed HBMs. Thus, the structure of HBMs in the absence and presence of melittin can be directly compared and questions about how deeply melittin penetrates into the bilayer, whether the ethylene oxide portion of the bilayer is hydrated, and whether melittin pores alter that hydration state can be addressed.

**Fig. 7:**
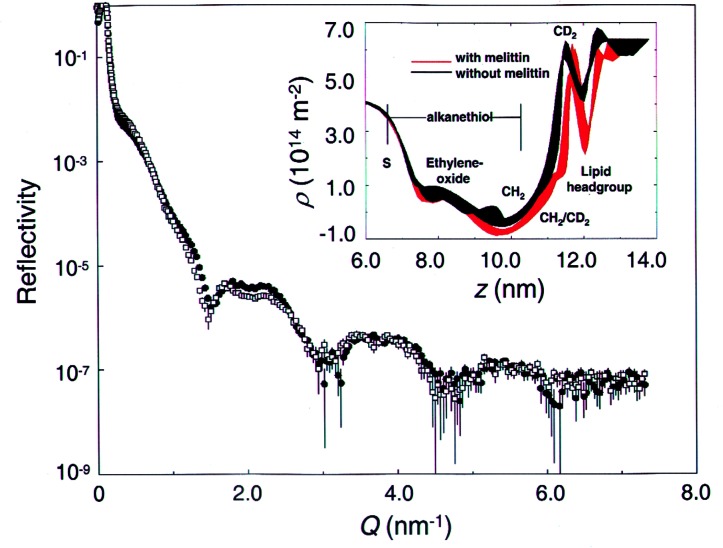
Neutron reflectivity data for THEO-C18/dDMPC hybrid bilayer membranes in D_2_O solution with (□) and without (●) melittin. The inset shows the boundaries of the families of scattering length density (SLD, *ρ*) profiles fitted with the model-independent fitting program PBS corresponding to the data obtained with (blue area) and without (black area) melittin, and identifies the positions of the components. The chromium and gold regions are not shown in the SLD profiles for clarity.

Neutron scattering length density (SLD) profiles along the z axis were determined from the reflectivity measurements using the model-independent fitting program PBS [25] developed at the NCNR. The SLD of each component of the bilayer is determined by its composition. Thus, a SLD profile is a representation of how the molecular composition of the bilayer changes as a function of *z*. Most notably, the SLD of H is an order of magnitude smaller than that of D, and is opposite in sign. Figure 7 shows the measured neutron reflectivity from THEO-C18 HBMs with and without melittin.

The corresponding fitted SLD profiles are shown in the inset. Note that the 5 nm gold layer that binds the sulfur moiety of the alkanethiol is not shown in the SLD profiles. It is evident from the SLD profiles that the ethylene oxide region (7 nm ≤ *z* ≤ 9 nm) of the THEO-C18 leaflet is not hydrated with D2O from the solution and that melittin does not alter the HBM in a way that allows bulk water to penetrate into this region. While the location of melittin appears to be primarily in the dDMPC headgroup region, the interfacial region between the alkane chains and the dDMPC acyl chains is displaced to higher *z* values by as much as 0.3 nm. This can only occur if the length of the alkane chains has increased, presumably through a decrease in tilt angle, indicating that melittin does have a significant effect on the THEO-C18 leaflet. These results agree with those of molecular dynamics simulations of a single melittin molecule in a DMPC bilayer [26], which suggest that the presence of melittin not only affects the lipid leaflet in which it is present, but affects the distal leaflet of the bilayer as well.

**Fig. 8:**
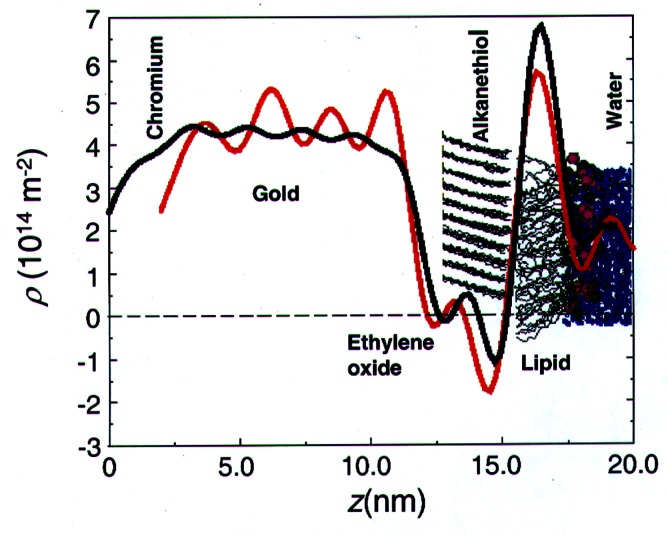
Scattering length density profile (red line) resulting from a direct inversion of the real part of the complex reflection amplitude compared with that predicted by a molecular dynamics simulation (black line) of a similar hybrid bilayer membrane. The picture derived directly from the molecular dynamics simulation shows the location of the significant features.

While the PBS fitting technique did allow determination of a range of SLD profiles for the HBMs with and without melittin, unambiguous inversion of reflectivity data to determine SLD profiles requires both the magnitude and phase of the reflected intensity. To measure the phase, reference layer methods, in which the film of interest is in contact with two or three reference films of known composition and thickness, have been employed [27,28]. Recently, an exact solution to the inversion problem has been found [29]; it requires only that two measurements of the film be made in which the “film surround,” i.e., either the fronting (incident) or backing (transmitting) medium surrounding the film, is varied. This technique was applied to the same THEO-C18 HBM system without melittin present. The resultant SLD profile is shown in Fig. 8, compared to the SLD profile predicted from molecular dynamics simulations of a similar HBM system [30].

### 3.6 Dynamics of Biological Materials

The recent commissionings of the Spin-Echo, High Flux Backscattering, and Disk Chopper Time-of-Flight Spectrometers allow access to a wide range of dynamic processes encompassing the 10^−7^ s to 10^−12^ s timescales. Along with the cold neutron SANS instruments, these spectrometers position the NCNR as the only U.S. neutron facility where both biological structure and dynamics studies can be performed, thus providing NIST with a unique probe of structure-function relationships in macromolecules and other biologically and pharmaceutically important systems.

A preliminary study of powdered lysozyme in glycerol is an example [31]. Differential scanning calorimetry measurements revealed an interesting behavior of lysozyme in the presence of glycerol: the addition of glycerol to *dry* lysozyme *lowers* the temperature at which protein unfolding occurs; but the addition of glycerol to *hydrated* lysozyme *increases* the protein unfolding temperature.

The lysozyme used for the neutron study was first treated with D_2_O to exchange most of the non-covalently bonded H with D; then the sample was dried. Since the remaining covalently-bonded H atoms have a much greater scattering cross-section than any of the other elements in the protein, neutron scattering provides a means of studying their motion, and thereby the motion *in situ* of the protein structures to which they are attached. Subsequent additions of D_2_O or deuterated glycerol began with this treated material.

Backscattering measurements analyzed to extract the mean square atomic displacement revealed a linear regime at lower temperatures characteristic of harmonic behavior. The atomic displacement breaks away above a dynamical transition temperature *T*_d_ to a more rapid rise with temperature, characteristic of anharmonic motion of the protein, a kind of behavior also seen in many disordered systems. In dry lysozyme this effect is absent, with harmonic behavior persisting up to the protein unfolding temperature.

The addition of glycerol to dry lysozyme has two distinct effects. As happens with the addition of D_2_O, it lowers the dynamical transition temperature, and increases the slope in the anharmonic regime above *T*_d_. However, below *T*_d_ the addition of glycerol (but not the addition of D_2_O) tends to *suppress* harmonic motion. This latter effect gives a clue to the behavior cited above: the suppression of harmonic motion by glycerol suggests that the protein may be inhibited from easily exploring regions in configuration space near the potential funnel where unfolding occurs. A backscattering exploration of dry lysozyme to which both D_2_O and glycerol are added systematically would be important in confirming this suggestion. Elucidating the roles of glycerol and water additions to protein motion can also provide the pharmaceutical industry with information useful for the better design of long-term storage of proteins.

## 4. Magnetism and Superconductivity

Magnetic neutron scattering originates from the interaction of the neutron’s spin with the unpaired electrons in the sample. The strength of this magnetic dipole-dipole interaction is comparable to the neutron-nuclear interaction, and thus there are magnetic cross-sections that are analogous to the nuclear ones that reveal the complete structure and full range of dynamics of materials over wide ranges of length and energy scales. Magnetic neutron scattering plays a central role in understanding the microscopic properties of a vast variety of magnetic systems, from the fundamental nature, symmetry, and dynamics of magnetically ordered materials to elucidating the magnetic characteristics essential in technological applications.

One traditional role of magnetic neutron scattering has been the measurement of magnetic Bragg intensities in the magnetically ordered regime. Such measurements can be used to determine the spatial arrangement and directions of the atomic magnetic moments, the atomic magnetization density of the individual atoms in the material, and the value of the ordered moments as a function of thermodynamic parameters such as temperature, pressure, and applied magnetic field. These types of measurements can be carried out on single crystals, powders, thin films, and artificially grown multilayers, and often the information collected can be obtained by no other experimental technique. For magnetic phenomena that occur over length scales that are large compared to atomic distances, the technique of magnetic SANS can be applied, in analogy to structural SANS. This is an ideal technique to explore domain structures, long wavelength oscillatory magnetic states, vortex structures in superconductors, and other spatial variations of the magnetization density on length scales from 1 nm to 1000 nm. Another specialized technique is neutron reflectometry, which can be used to investigate the magnetization profile in the near-surface regime of single crystals, as well as the magnetization density of thin films and multilayers, in analogy with structural reflectometry techniques. This particular technique has enjoyed dramatic growth during the last decade due to the rapid advancement of atomic deposition capabilities.

Neutrons can also scatter inelastically, to reveal the magnetic fluctuation spectrum of a material over wide ranges of energy (from about 10^−8^ eV to 0.25 eV) and over the entire Brillouin zone. Neutron scattering plays a truly unique role in that it is the only technique that can directly determine the complete magnetic excitation spectrum, whether it is in the form of the dispersion relations for spin wave excitations, wave-vector and energy dependence of critical fluctuations, crystal field excitations, magnetic excitons, or moment fluctuations. In the present overview we will discuss a few examples of work carried out at the NCNR.

### 4.1 Magnetic Superconductors

**Fig. 9:**
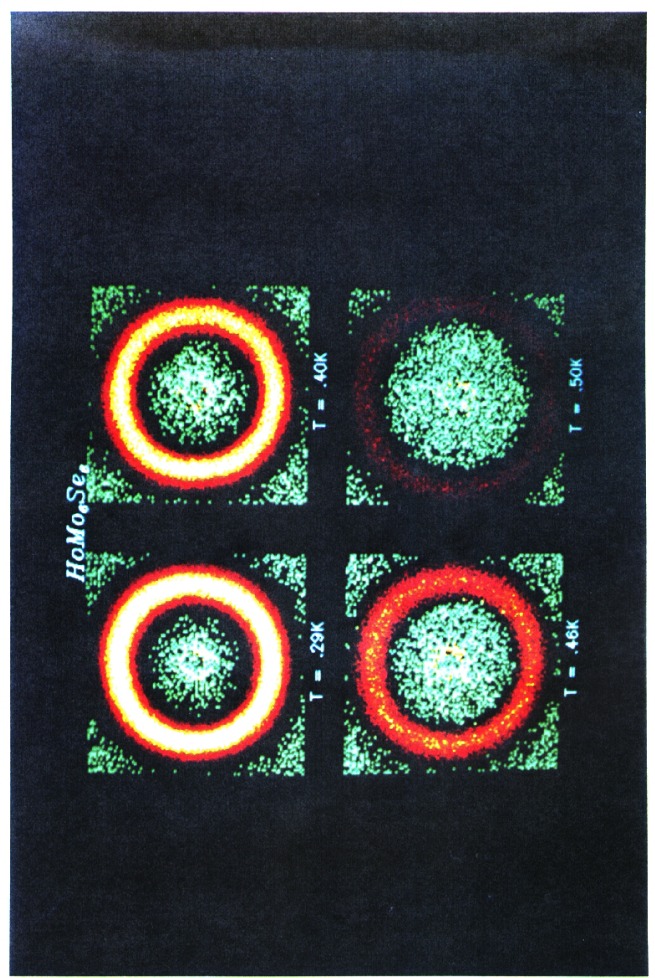
Magnetic Bragg scattering from a powder of the superconductor HoMo_6_Se_8_. The scattering would be centered in the four images if the magnetic order were allowed to be purely ferromagnetic, but the superconductivity forces the magnetic structure to oscillate on a length scale of about 10 nm, producing the ring of scattering.

The effects of magnetic impurities and the possibility of magnetic ordering in superconductors have had a rich and interesting history. Neutrons have played an essential role in determining the nature of the magnetic order, since the Meissner screening of the superconducting electrons masks the magnetism from most probes. The first examples of true long-range magnetic order coexisting with superconductivity were provided by the ternary Chevrel-phase superconductors (*R*Mo_6_S_8_) and related (*R*Rh_4_B_4_) compounds (where *R* is a specific rare earth ion for each compound). The surprising occurrence of superconductivity suggested that the magnetic rare earth ions were somehow isolated from the superconducting electrons. The magnetic ordering temperatures were all low, ≈1 K, and thus it was argued that electromagnetic (dipolar) interactions should dominate the energetics of the magnetic system. For most materials antiferromagnetism is favored, and the magnetization averages to zero on the length scale of a unit cell, resulting in a weak influence on the superconducting state. In the rare and more interesting situation where the magnetic interactions are ferromagnetic, there is strong coupling to the superconducting state that originates from the internally generated magnetic field. Figure 9 shows the magnetic scattering for HoMo_6_Se_8_, which becomes superconducting at 5.5 K, and then tries to order ferromagnetically at lower temperature. The beam center is in the middle of each image, which is where a truly ferromagnetic peak would be observed. However, the competition between the superconducting order parameter and the ferromagnetic order gives rise to a long wavelength oscillatory magnetic state as shown in the SANS image [32]. This is just a powder diffraction ring of scattering with a *d* spacing of ≈10.0 nm, taken with a two-dimensional position sensitive detector. We see that the strength of the scattering increases with decreasing temperature, while the size of the ring decreases as the system tries to push closer to a ferromagnetic state. However, the ferromagnetic energy is never large enough to quench the superconducting state, which persists to low temperature. In the related HoMo_6_S_8_ material the superconductivity is weaker (*T*_C_ = 1.8 K), and the material locks into ferromagnetism at low *T* as the superconducting state is destroyed. More recently the magnetic ordering has been investigated in the *R*Ni_2_B_2_C class of superconductors [33], where the magnetic ordering temperatures are much too high to be explained by dipolar interactions and there is a clear competition with the superconductivity. Finally, we note that these magnetic superconductors have generated renewed interest with the discovery of the mixed ruthenate-cuprate RuSr_2_GdCu_2_O_8_ system, where the Ru orders at 135 K with a ferromagnetic component in the magnetic structure, while superconductivity occurs at 30 K [34].

### 4.2 High *T*_C_ Superconductors

Nowhere is the magnetism of more interest than in the cuprate superconductors, which offer new and interesting perspectives into our understanding of “magnetic superconductors” [35]. The central feature that controls all the cuprate oxide materials is the strong copper-oxygen bonding, which results in a layered Cu-O crystal structure. In the undoped “parent” materials this strong bonding leads to an electrically insulating antiferro-magnetic ground state. The exchange interactions within the layers are much stronger than between the layers, and typically an order-of-magnitude more energetic than the lattice dynamical energies. The associated spin dynamics and magnetic ordering of the Cu ions is thus driven by this two-dimensional (2D) nature, and the importance of low-dimensional magnetism in the cuprates has spurred general interest of magnetic systems in restricted geometries [36]. With electronic doping, long range antiferromagnetic order for the Cu is suppressed as metallic behavior and then superconductivity appears (up to 160 K), but strong antiferro-magnetic spin correlations still persist in this regime. It is this large magnetic energy scale that is associated with the high superconducting transition temperature and exotic, d-wave superconducting pairing, as has been inferred via vortex flux lattice measurements [37].

**Fig. 10:**
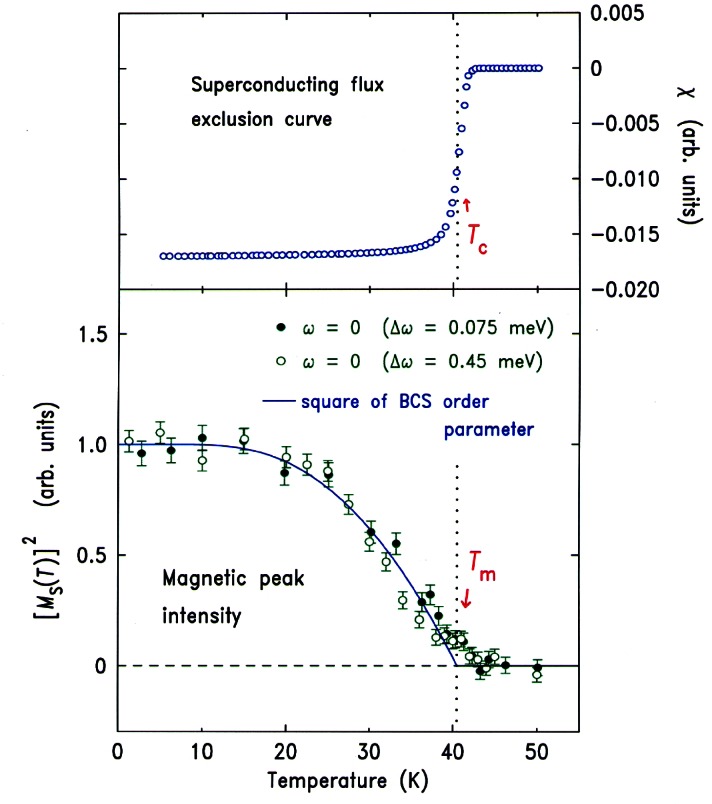
(a) (top panel) Zero-field cooled magnetic susceptibility in arbitrary units, indicating a superconducting transition of 42 K. (b) (bottom panel) Intensity of the incommensurate magnetic Bragg scattering as a function of temperature. The solid curve represents the BCS superconducting order parameter.

The prototypical cuprate superconductor is YBa_2_Cu_3_O_7_, and the magnetic response in the superconducting state is surprisingly simple, consisting of a sharp magnetic mode at an energy of 41 meV at the zone boundary of the CuO_2_ square lattice. The resonant excitation disappears in the normal state (at optimal doping) and must be thought of as a novel collective mode of the superconductor [38]. An even more interesting combination of cuprate magnetism and superconductivity is exhibited by the single layer La_2_CuO_4+δ_ material, where the extra oxygen δ that dopes the system orders in stages. Figure 10a shows the superconducting shielding signal measured after cooling in zero field [39]. The transition is sharp with an onset *T*_C_ = 42 K, the highest of any 2-1-4 system. The interesting thing here is that long range magnetic order of the Cu moments is also observed in this material. The intensity of the magnetic Bragg peak is shown in Fig. 10b as a function of temperature, where it is seen that the onset of the magnetic scattering occurs at the same transition temperature as superconductivity. The solid curve is the BCS order parameter curve, and the agreement demonstrates that the magnetic order and superconductivity are inexorably linked.

The nature of the cooperative magnetic states and their corresponding spin excitations in amorphous metallic magnets has been the subject of many investigations at the NCNR, due both to the wide range of interesting physics these systems exhibit and to the broad range of applications for these materials [42]. An amorphous material is by definition globally isotropic, by which we mean that there is no preferred direction in the material. This requires that the energy of the excitation, whether it is “lattice” dynamical, electronic, or magnetic in origin, depends only on the magnitude *q* of the wave vector ***q***.^4^ The classification of amorphous magnets then falls into two general categories, depending of the nature of the local anisotropy. In rare earth systems, for example, the single-site crystal field anisotropy is large compared with the exchange energy and varies randomly in magnitude and direction from site to site. This has the effect of severely disrupting the exchange interactions in the material and consequently lowering the effective interactions below the lower marginal dimensionality, eliminating the possibility of any long range ordering phenomena to occur. These systems offer the additional advantage that the microscopic interaction parameters can be determined from their crystalline counterparts, facilitating comparison between theory and experiment. The magnetic properties for this class of materials are found to be typical of spin glasses, and have proved to be interesting examples of random field systems.

The case when the anisotropy is weak compared to the exchange interaction, such as for most transition metal based glasses, allows long-range ferromagnetic order to develop, and indeed such materials now provide the best known examples of isotropic ferromagnets. For any isotropic ferromagnet the nature of the low temperature excitations at long wavelengths is well established; in this hydrodynamic or continuum limit the small wave vector excitations are the familiar spin waves or magnons, and the theory works well over a surprisingly wide range of temperatures. Hydrodynamic theory can also be employed in the vicinity of the Curie temperature *T*_C_, and the critical dynamics are found to follow the conventional behavior for the universality class of a three-dimensional (D = 3) isotropic (*n* = 3) ferromagnet. At larger wave vectors, however, the inherent randomness of the system must dominate the physics. This will change the nature of the excitations, and generally one must think of these in terms of a general density of excitations rather than in terms of well-defined excitations that obey a dispersion relation.

One of the noteworthy advantages of amorphous systems is that they can generally be fabricated over a wider range of compositions than the corresponding crystalline systems since there is no requisite for thermodynamic equilibrium. This allows one to better control and tailor the magnetic properties, both in the investigation of physical phenomena of interest as well as when developing a material for a specific application. Fabrication techniques such as rapid quenching from the melt tend to minimize clustering and chemical short-range order, which often is a problem in crystalline alloy systems, and this can simplify the interpretation of measurements and thereby clarify the underlying physics of interest. Hence amorphous magnets have been chosen for a variety of studies as a function of magnetic concentration, in particular in the low concentration regime where the network of spins is highly ramified and long range order is becoming disrupted. This leads to phenomena such as reentrant ferromagnetic order, frustration, transverse freezing, and spin glass behavior. Neutron inelastic scattering is the ideal probe of the dynamical behavior of magnetic systems and elucidates the fundamental behavior of these prototypical magnets. In the long wavelength (small *q*) hydrodynamic limit there is a “Goldstone mode” with dispersion relation given by the familiar expression relating energy *E*_sw_ and excitation wavevector *q* through the stiffness coefficient *D* at temperature *T* : *E*_sw_ = *D*(*T*) *q*^2^. Most of the transition-metal amorphous magnets are in fact excellent approximations of isotropic ferromagnets. The quantitative value of *D* depends on the details of the interactions and the nature of the magnetism, such as whether the magnetic electrons are localized or itinerant, or the structure is amorphous or crystalline, but the general form of the spin wave dispersion relation, and hence the spin wave density of states, is invariant. Measurements have also been carried out to investigate the behavior of the magnon linewidths as a function of behavior, both at low temperatures and in the critical regime, and these soft amorphous magnets turn out to be prototypes of isotropic ferromagnets [42].

### 4.3 Colossal Magnetoresistive Oxides

The recent discovery of huge magnetoresistance effects in the manganese oxide class of materials (such as La_1−_*_x_* A*_x_*MnO_3_, where A = Sr, Ca, or Ba) has rekindled intense interest in these systems for two reasons. First and foremost is that the colossal magnetoresistance (CMR) offers enormous potential for technologies such as read/write heads, sensors, and spin-polarized electronics. The other reason is that the charge, spin, and lattice degrees of freedom are strongly coupled together, leading to a delicate balance of interactions that gives rise to a rich variety of physical phenomena of current interest in condensed matter science. These include a metal-insulator transition that is juxtaposed with ferromagnetism, charge ordering, orbital ordering, polaron formation, electronic phase separation, and spin and charge stripes. The underlying similarities with the cuprate oxides express a commonality of many of the materials properties and underlying physical concepts in this oxide class of systems. Recent progress in our understanding of the cuprates has provided insights into the manganites, and a deeper understanding of the fundamental properties of the manganites will surely elucidate the shared concepts underlying both classes of materials.

**Fig. 11:**
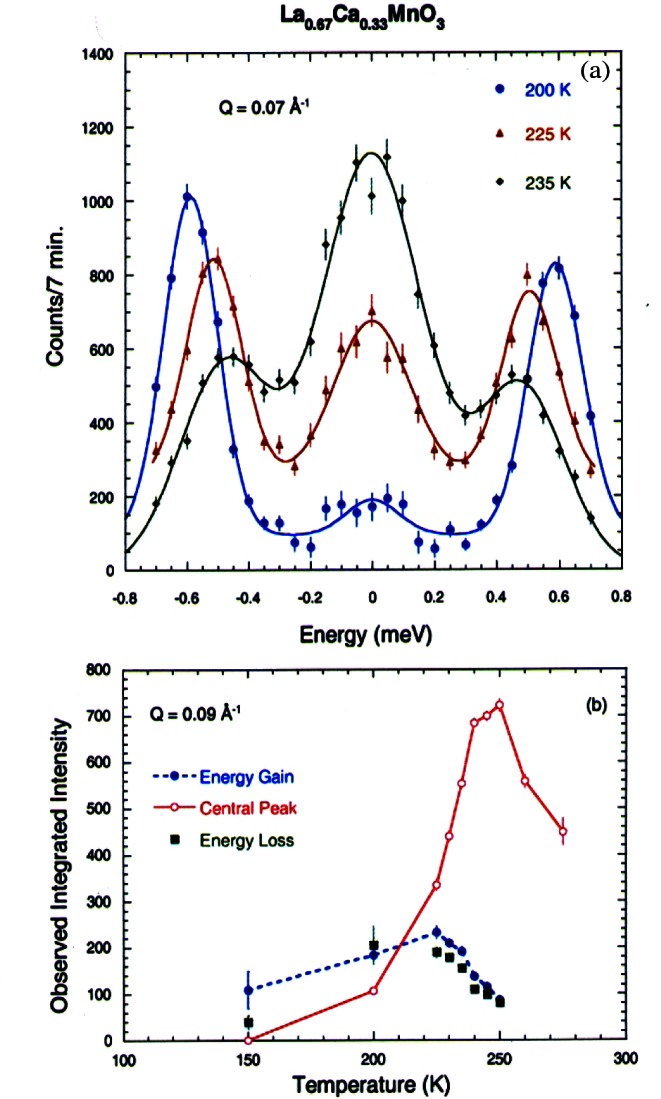
(a) Inelastic scattering at a wave vector of 0.7 nm^−1^. At 200 K the spectrum is dominated by spin waves in energy gain and energy loss, while as *T*→*T*_C_ the spin waves lose strength while the quasi-elastic scattering rapidly develops and dominates the magnetic fluctuation spectrum, as shown in (b).

The largest CMR effects are for the Ca-doped system, which is ferromagnetic for 0.15<*x*<0.5 [40]. The ground state magnetic excitations were found to be conventional spin waves as shown in Fig. 11a, with a gapless dispersion relation *E*_SW_ = *D*(*T*) *q*^2^ [41]. This demonstrates that these manganites are “soft” ferromagnets, comparable to the very soft amorphous ferromagnets [42]. At elevated temperatures, however, the spin waves were found not to renormalize as expected, and in particular they did not collapse at the ferromagnetic Curie temperature. Instead, a quasi-elastic spin-diffusion component in the fluctuation spectrum develops as shown in Fig. 11a, and becomes the dominant spectral weight as *T*→*T*_C_ (Fig. 11b), while the spin waves actually decrease in spectral weight. This is in stark contrast to the conventional behavior observed for isotropic ferromagnets. The magnetic correlation length is also anomalously small and only weakly temperature dependent, instead of diverging at *T*_C_. These results, together with single crystal diffraction measurements that reveal the development of lattice polarons as *T*→*T*_C_ in this and related manganites [43,44], have demonstrated that the anomalous central peak, the resistivity, and the lattice polarons have a common origin.

### 4.4 Magnetic Multilayers and Films

**Fig. 12:**
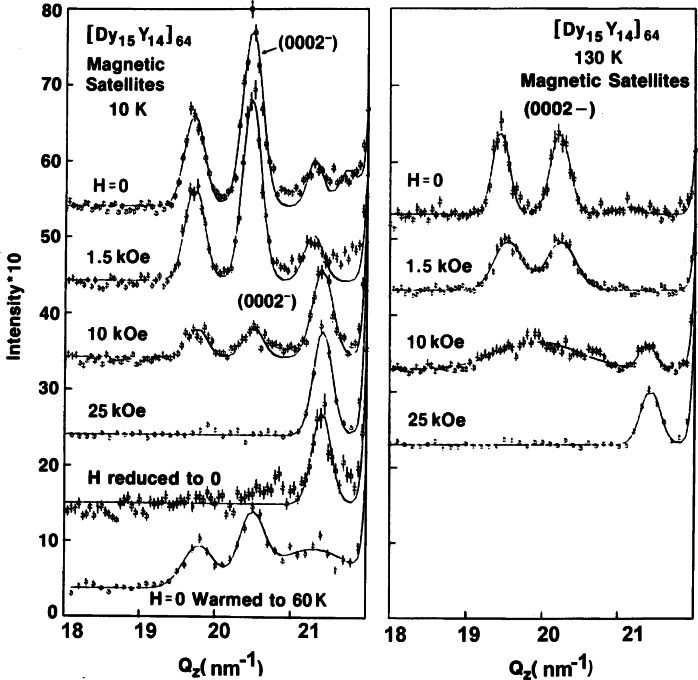
The left side of the figure shows the magnetic field dependence of the neutron diffraction peaks in a superlattice consisting of alternating layers of 15 atomic planes of dysprosium with 14 atomic planes of yttrium. The magnetic superlattice peaks disappear as the magnetic field converts the dysprosium helix to a ferromagnet. On the right side of the figure, at higher temperatures, the magnetic field breaks the coupling between helices in the separate dysprosium layers, leaving a broad peak centered at the dysprosium helix wave vector.

In recent years, composite and nanoscale structures have been at the center of many advances in materials’ properties and devices. Magnetic thin films and multi-layers are examples of such structures and have been extensively studied at the NCNR by neutron diffraction and reflection using polarized beams [45, 46, 47]. Neutron diffraction measurements on rare-earth multi-layers, for example, represent some of the very earliest work showing that exchange coupling information can be transmitted between magnetic layers through surprisingly thick non-magnetic layers. Figure 12 shows neutron diffraction scans of the magnetic peaks in a film where each group of 15 atomic planes of magnetic dysprosium is separated by a group of 14 atomic planes of non-magnetic yttrium, and then this basic bilayer is repeated [48]. Multiple peaks are observed as a result of the superlattice structure of the film, and this implies that the dysprosium helical magnetic structure must be coherent across multiple non-magnetic yttrium layers. The relatively weak interlayer coupling can also be readily controlled by modest magnetic fields, as shown in the figure. The right side of the figure shows how the breakdown of the coherence across the non-magnetic layers leads to the disappearance of the magnetic superlattice peaks.

Similar exchange coupling has been observed more recently in transition-metal oxide multilayers, and these materials are now being used in a variety of applications such as high-sensitivity magnetic sensors and read/write heads [49]. In these systems, a film of high-anisotropy magnetic material is grown on top of an isotropic ferromagnet, producing an exchange-biasing. A suitably engineered system can exhibit weak uni-directional anisotropy, a property finding applications in magnetic switching. The research at the NCNR has lead to an to an understanding of the magnetic interactions responsible for this exchange-bias phenomenon.

The cold neutron instrumentation that has been developed in recent years at the NCNR is unmatched in the United States and is competitive with the best in the world. These new neutron spectrometers have dramatically improved our measurement capability for exploring the properties of both magnetic and superconducting materials. Presently we are developing new thermal neutron instrumentation that will also be unparalleled in this country, and we anticipate that these new instruments will produce an equally important impact on future investigations of magnetic and superconducting phenomena.

## 5. Neutron Diffraction From Materials

### 5.1 Neutron Crystallographic Instruments

Neutrons have a number of advantages and disadvantages compared to x-rays for crystallographic characterization of materials. While atoms with the largest number of electrons typically dominate x-ray scattering from a material, the strength of neutron scattering is a function of isotope species and varies irregularly across the periodic table. For many materials, neutrons provide much better sensitivity to establishing the location of lighter atoms in the unit cell. Since the cross section for scattering a neutron from a nucleus is not a function of the scattering angle, neutrons are typically far better able to differentiate between site occupancies and atomic displacement parameters. Thus, neutrons are the probe of choice for elucidating site sharing or for vacancy determination. Finally, neutrons are far more penetrating than x rays, allowing the bulk of a sample to be probed in contrast to x-ray experiments that are usually surface-sensitive.

#### 5.1.1 The 1960s and 1970s

The value of neutrons for crystallographic characterization was one of the driving forces for the construction of the NBSR reactor. Both a powder and single-crystal diffractometers were among the initial complement of instruments installed when the reactor first began routine operations in 1969. The original powder diffraction instrument was built at Brookhaven National Laboratory to the specifications of scientists from the Naval Ordnance Laboratory, whose primary interest was magnetic materials. This instrument was located at beam tube 1 (BT-1). It had a single detector and a conventional drum-type monochromator, with a continuously variable monochromator takeoff angle (2*θ*_mono_). The original single-crystal instrument, which was located at BT-8, was a conventional four-circle diffractometer. Most components for this instrument were brought to NBS from the Naval Research Laboratory. With only a single detector, intensities of Bragg reflections could only be measured one at a time. To get useful data in a reasonable amount of time, good quality crystals with a volume of at least several cubic millimeters were required. This created a severe limitation on the range of problems that could be usefully studied.

The highest monochromator diffraction angle that was feasible with the original BT-1 powder instrument was about 75°, which is sufficient for many important problems, but not ideal. Some structural studies were carried out with the single detector instrument [50]. It was quickly evident that the powder method was well adapted to the use of multiple detectors, which would markedly improve the utility of the instrument. The second-generation BT-1 instrument was constructed by replacing the single detector in the initial instrument with a detector assembly having five detectors with nominal spacing of 20° in 2*θ*. This five-detector instrument, which became routinely operational in 1978, allowed a powder pattern with a range of 100° to be collected with only a 20° scan. Typical data collection times were two days per scan. The instrument was still limited by the maximum monochromator takeoff angle of 75°, but the moderately high resolution made it extremely powerful for studies of a large class of technologically important materials. Data from the five-detector instrument was the basis for much important science, notably the first demonstration that the superconductivity of YBa_2_Cu_3_O_6+δ_ was extremely sensitive to the oxygen content parameter δ [51]. The instrument was also extremely powerful for studies of a large class of technologically important materials, such as the zeolite RHO [52].

The development of the full-pattern fit method by H. Rietveld in 1969 greatly extended the crystallographic utility of powder diffraction through the technique now known as Rietveld analysis [53]. The value of this work was recognized quickly at NBS and within a few years a version of Rietveld’s code was brought over from Harwell, U.K., and installed on the NBS mainframe computer of the time. Another advance in data analysis techniques was the use of rigid-body constraints to model thermal motion in organic molecules. In the 1960s, the elements of the rigid-body motion tensors had been used to describe the motion in refined structures [54], but in the 1970s the first application of these tensor elements as the independent parameters in a structure refinement was developed by NBS for the study of methyl group libration in 1,2,4,5 tetramethyl benzene (durene) [55]. This study showed that constrained refinement could be a powerful tool in structure studies. Another major advance in crystallographic analysis was made at NBS during this same period, with the development of a robust method to uniquely determine a reduced cell [56]. This offered a powerful method for the classification of materials independently of the details of the material symmetry. Later work at the NCNR greatly expanded the utility of the reduced cell for structural analysis and for crystallographic database searching [57–59].

Also, during the 1970s it became evident that much of the most significant structure work lay in the field of biological macromolecules, and that useful studies in this field would require a major improvement in data collection rates. At that time, area detectors for neutrons with sufficient angular range and resolution for macromolecular crystallography were beyond the state of the art, but linear position-sensitive detectors up to one meter long with resolutions of a few millimeters had become available. To exploit this technology, in the late 1970s the four-circle diffractometer was moved to a satellite position on BT-7, and the BT-8 instrument was equipped with a linear PSD lying in a vertical plane, making use of the flat-cone Weissenberg geometry [60]. Early work in neutron single-crystal diffraction produced structure determinations of metallo-organic complexes [61], minerals [62], explosive materials [63], and materials containing both hydrogen and heavy metals [64]. The later BT-7 flat cone diffractometer was used for several ground-breaking studies of protein structures, most notably a refinement of the structure of bovine pancreatic tripsin inhibitor [2].

#### 5.1.2 The 1980s to the Present

During the 1980s it was apparent that important science required a further upgrade of powder diffraction capability, but that this could only be achieved by a major redesign and reconstruction. It was also apparent that the existing instrument was well adapted to studies of large classes of materials, so that any new instrument must be at least as well adapted for those materials. On the other hand, other classes of materials required higher resolution, particularly for Bragg reflections with small *d*-spacings. All measurements would benefit from substantially higher data rates.

The conceptual design for the third-generation BT-1 neutron powder diffractometer was complete by 1984, but fiscal constraints and some engineering problems, particularly with the fabrication of suitable collimators, delayed its completion until 1992. Design specifications for the new BT-1 required that the instrument response function have strictly Gaussian peak broadening, that this resolution function be selectable according to the needs of the study, and that data rates should be as high as reasonably feasible. The new instrument must also outperform the second-generation instrument.

**Fig. 13:**
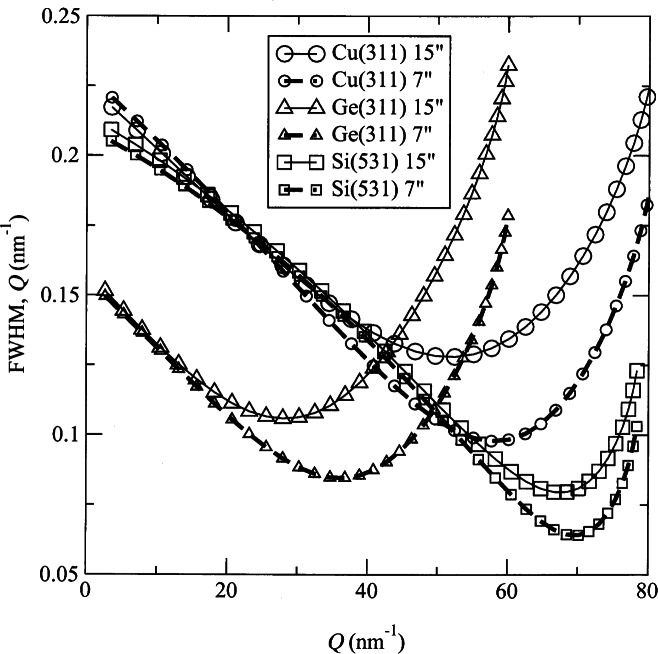
The resolution of the BT-1 powder diffractometer that can be obtained for various combinations of monochromators and in-pile collimators (indicated in the inset in minutes of arc.) These values were obtained from the fits to observed data.

The present 32-detector BT-1 powder diffractometer has proven highly versatile. The instrument resolution choices are shown in Fig. 13. For *Q* values above ≈30 nm^−1^ (2*θ*>45° at *λ* = 0.154 nm), the instrument offers the highest resolution of any neutron powder diffractometer in the United States. Data collection times vary depending on the sample size, the desired resolution, the amount of non-Bragg background scattering from the sample and its mounting, and the signal-to-noise level acceptable for intended measurement. Under optimal conditions complete diffraction measurements are made in 2 to 3 h and the average data collection measurement requires 6 to12 h. The instrument is used to collect approximately 1000 complete diffraction patterns every year, for a wide range of problems in physics, chemistry, and materials science.

Software development also continues to flourish at the NCNR. Work on an extremely powerful and adaptable neutron Rietveld code, REFINE, specifically designed for multiple-detector neutron powder instruments has continued. The NCNR has also created EXPGUI, a graphical user interface for the popular GSAS package of Los Alamos. This interface has significantly decreased the time it takes to learn use of this complex crystallographic package. A novel visually-based approach to powder diffraction indexing, implemented in the CMPR program, allowed DuPont to determination unit cell parameters for triclinic pharmaceutical materials based on synchrotron and electron diffraction data [65].

#### 5.1.3 Neutron Crystallographic Structural Determination: Examples

The majority of structural determinations performed using powder diffraction data are for metal oxide materials, with applications to fields of interest such as negative thermal expansion [66,67], high-dielectric ceramics [68,69], CMR materials (see section 4.3) and superconductivity. The study of perovskite and pyrochlore oxide materials is also an important area, due to properties such as magnetorestriction [70], charge disproportionation [71], and ionic conductivity [72].

##### 5.1.3.1 Colossal Magnetoresistive Materials

**Fig. 14:**
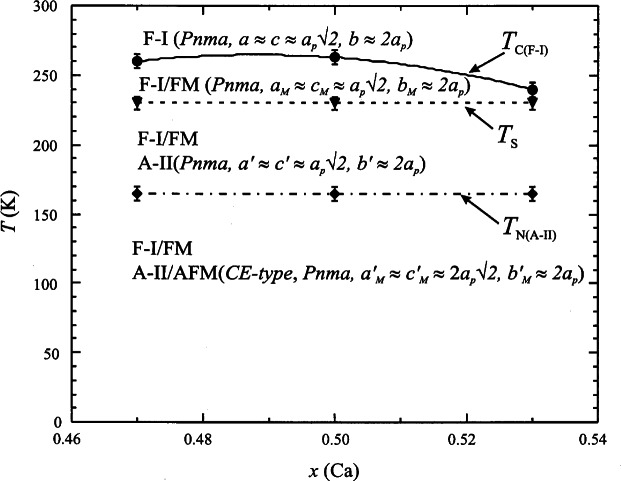
Phase diagram of La_1−_*_x_* Ca*_x_*MnO_3_ in the range of compositions 0.47 ≤ *x* ≤ 0.53. The horizontal curves separate, going from top to bottom: (i) the ferromagnetic (FM) transition of the high-temperature phase (F-I) at ≈260 K; (ii) the formation of the low-temperature A-II phase, which appears at ≈230 K; and (iii) the antiferromagnetic (AFM) transition which occurs in the A-II structure at the Nel temperature *T*_N_ ≈ 160 K. As shown in the diagram, the ferromagnetically ordered F-I phase and the antiferromagnetically ordered F-II phase coexist at low temperature.

**Fig. 15:**
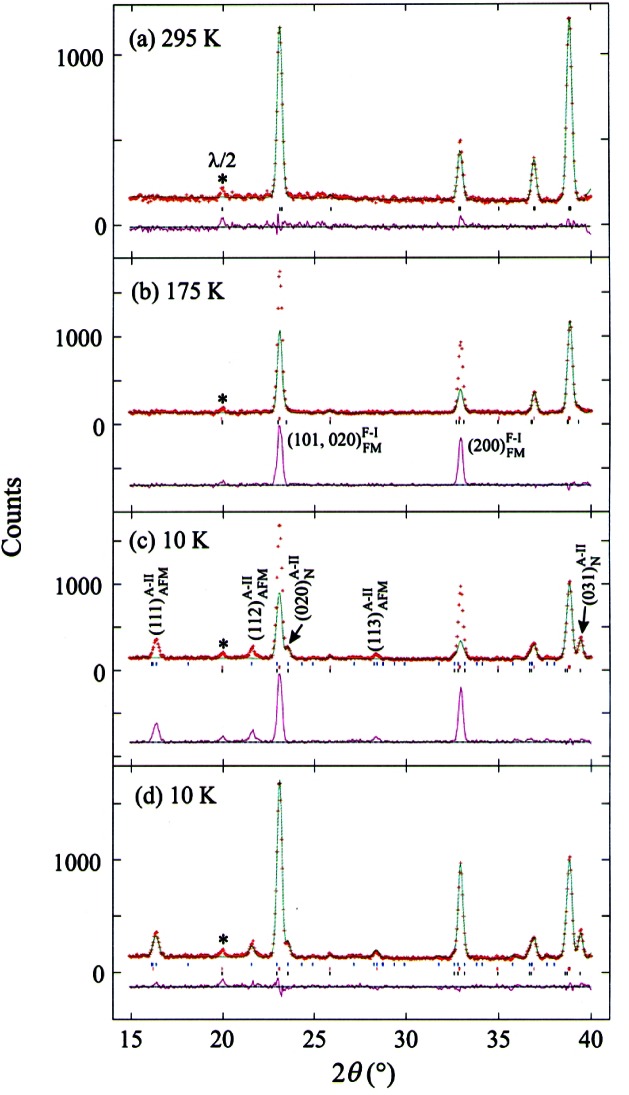
Neutron powder diffraction pattern of the = 0.47 composition in the angular range between 15° and 40° at various temperatures, employing the Cu(311) monochromator. In (a) only the nuclear peaks of the F-I phase are present. (b) The ferromagnetic reflections associated with the F-I phase are indexed in the difference plot, obtained by subtracting from the observed intensities the calculated profile. Note that at 175 K only the magnetic and nuclear peaks of F-I are clearly visible, since the fraction of the A-II phase is small and peaks are broad at this temperature. (c) At 10 K both the ferromagnetic peak of F-I phase and the antiferromagnetic peaks of A-II phase contribute to the diffraction pattern, and these peaks are clearly visible in the difference plot. Note that the ferromagnetic peaks at 10 K appear at the same angular positions as those observed at 175 K. (d) Complete fit of the neutron pattern, taking into account all nuclear and magnetic intensities. The small peak at 2*θ* ≈ 20° is the second order of the strongest nuclear reflection.

An example of a CMR material displaying the intricate interplay among magnetic, structural and electronic properties is the doped perovskite La_1−_*_x_* Ca*_x_* MnO_3_ (*x* = 0.47, 0.50, and 0.53) [73]. Powder diffraction at BT-1 was used to elucidate the phase separation, structure, and magnetic ordering of these compounds. Above 265 K the materials are insulating paramagnets in the space group *Pnma.* Below this temperature they become *Pnma* ferromagnetic conductors, and at ≈230 K there emerges a coexisting distinct second insulating crystallographic phase with the same space group. At 160 K this second phase orders antiferromagnetically with a CE-type magnetic structure (see Ref. [73] for references describing this complicated structure.) The conducting ferromagnetic and insulating antiferromagnetic phases coexist in intimately mixed small domains down to 5 K, the lowest temperature measured. Figure 14 shows the central result of the study, and Fig. 15 displays the relevant diffraction patterns and their interpretations.

##### 5.1.3.2 Competition Between Magnetism and Superconductivity

**Fig. 16:**
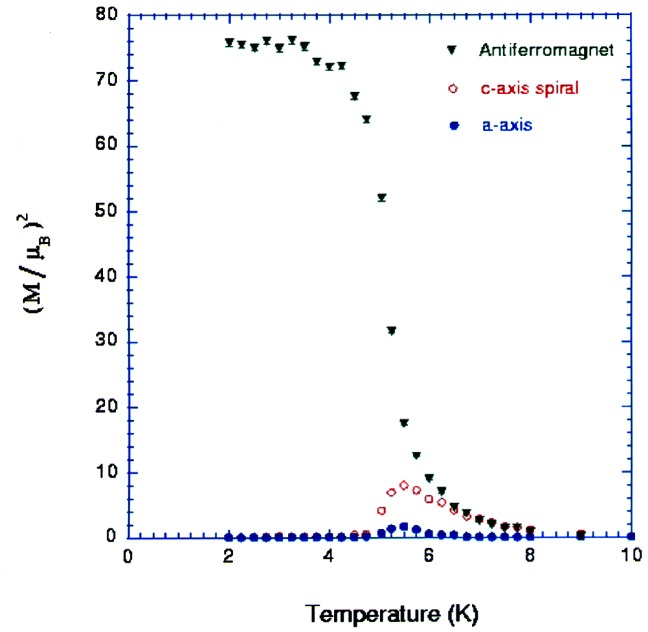
Square of the sub-lattice magnetizations (proportional to the scattered intensities) of the antiferromagnetic, *c*-axis spiral, and *a*-axis magnetic peaks as a function of temperature for HoNi_2_B_2_C. The intensities for the incommensurate magnetic peaks are suppressed below the temperature where the superconducting state reemerges (≈5 K).

The competition between magnetic ordering and superconductivity has also been the subject of series of crystallographic investigations at NIST in several systems. An example (also mentioned in section 4.1) is the *R*Ni_2_B_2_C system with *R* = Y, Ce, Pr, Nd, Tb, Dy, Ho, Er, Tm, or Yb [74]. While the compounds of this system have *I4/mmm* crystal structure symmetry over the full range of compositions and temperatures studied, they display many forms of magnetic ordering, centering on the rare-earth atoms. There are examples of commensurate and incommensurate antiferromagnetic ordering, spiral antiferromagnetism, and spin-density wave states. The case of the Ho compound at low temperatures is particularly interesting since it displays the coexistence of two forms of antiferromagnetic ordering: a commensurate one coexisting with superconductivity and an incommensurate one that competes with superconductivity. Figure 16 displays the intensity of the magnetic diffraction peaks as a function of temperature showing these effects.

##### 5.1.3.3 Determination of Minority Phases

**Fig. 17:**
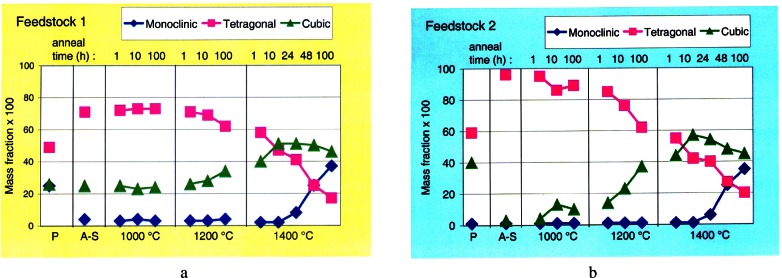
Change in phase compositions for two different feedstocks of yttria stabilized zirconia coatings upon annealing. P is the starting feedstock powder; A–S is the as-sprayed coating. The annealing times at three different temperatures are indicated on the graphs. Note the convergence of mass fractions in graphs (a) and (b) of the coatings annealed for 100 h at 1400 °C.

Crystallographic modeling of powder diffraction data has served very well for determination of phase composition. One example of this work has been a series of detailed studies of the degradation of yttria-stabilized zirconia thermal barrier coatings as a function of temperature and time (see Fig. 17) [75]. This work has demonstrated the fundamental difficulties in using this class of materials for this purpose. x-ray diffraction, restricted by a small penetration depth and by low sensitivity to scattering from light atoms, is very limited in its ability to analyze such materials.

##### 5.1.3.4 Zeolites

Microporous materials continue to be a major focus of structural research within the NCNR. Examples are zeolitic materials with the RHO framework. A recent study demonstrated that unusual cation siting could be understood in terms of the coordination requirements of the alkali metal cations [76]. Combined neutron and synchrotron x-ray measurements have been demonstrated to be the most conclusive method for cation site determination in these materials [77]. This combined method can also be used to find locations and orientations of complex organic molecules in the pores of zeolitic materials [78].

### 5.2 Engineering Applications of Neutron Diffraction

#### 5.2.1 Crystallographic Texture Measurements

Two Department of Defense guest scientist groups that started work at the NBSR in the early 1970s drove interest in use of neutron diffraction for engineering measurements. One group was from the Navy with primary interest in magnetic materials, and the other from the Army with primary interest in energetic materials. The Army group, from the Picatinny Arsenal, began to expand their program in the mid-1970s to include some metallurgical problems. Among these was a question of quality control of shear-spun shaped-charge liners. Specifically, texture in copper shaped-charge liners was correlated with performance using measurements from the BT-6b single-crystal diffractometer. The use of neutrons for “conventional” texture measurements continued through the 1980s and 1990s. In particular, applications of the technique to metallurgical specimens of interest to the Army and Navy were dominant. Both the Army and Navy used shaped-charge liners for armor penetration applications. As with the copper liners mentioned above, texture in other liners played a role in the mechanical properties and how the liner collapsed, and ultimately in performance. In heavy metal liners such as those of depleted uranium [79, 80, 81] and tantalum [82] x rays might penetrate only a few micrometers of material so that true bulk texture was very difficult to obtain by x-ray methods. Even for a less heavy metal like molybdenum, the use of neutron diffraction provided a relatively straightforward way of determining texture [83].

#### 5.2.2 Depth Profiling of Residual Strain

Depth-profiling of residual strain by neutron diffraction became important at NBS in the early 1980s, using this BT-6 triple axis spectrometer. Details of the technique and a review of recent literature are presented in Ref. [84]. At this time, the Army was concerned about residual stress produced in depleted uranium kinetic energy (KE) penetrators when rotary straightening was used in the fabrication sequence. KE penetrators were utilized in about half of the Army’s armor-piercing munitions. As mentioned above, x-ray diffraction—already a well-established technique for the determination of residual stress on surfaces—could not penetrate a grain of a heavy metal. This made it unusable for stress determination. Army engineers were forced to resort to the Sachs boring-out technique, which was expensive, of limited availability, and destructive.

In contrast to x rays, uranium was more transparent than steel to neutrons in the diffraction wavelength regime (≈0.1 nm). The first use of neutron diffraction for the determination of residual stress in a KE penetrator alloy, U with a Ti mass fraction of 0.75 %, was made successfully at the NBSR [85]. This was followed by a series of studies of residual stress distributions in K.E. penetrators in which fabrication and processing conditions were varied in order to improve mechanical properties and performance. One rather dramatic result of these studies was to show the effect of cold work on the residual stress distribution. Even a 7 % reduction-in-area swaging reverses the hoop stress near the surface from ≈300 MPa compressive to ≈100 MPa tensile [86,87]. This was highly undesirable in that uranium was susceptible to stress corrosion cracking which is inhibited by compressive surface stresses.

Residual stress projects in the 1990s included the determination of stresses under the threads of aluminum components (“ogives”) made by two slightly different fabrication processes: one of which worked (“A”), the other of which occasionally didn’t (“B”). Neutron diffraction revealed clearly tensile axial stresses in the B-type in the failure region. The A-type ogives showed compressive stresses in the same region [87].

#### 5.2.3 An Improved Instrument for Engineering Applications: DARTS

The need for better measurements inspired the design of a new instrument, tailored to metallurgical needs, but that also could be used for single crystal measurements. This instrument, the double axis spectrometer for residual stress, texture, and single crystal analysis (DARTS), was completed in 1996, displacing the single-crystal instruments at BT-6 and BT-8. The DARTS instrument has three monochromators: graphite, Cu(220), and Cu(311), which allow wavelengths from 0.09 nm to 0.58 nm to be used. This flexibility allows the wavelength to be selected for most reflections so that 2*θ* is near 90°. The use of 90° instrument geometry minimizes the volume of sample where diffraction is measured, called the gauge volume. Where possible, the monochromator takeoff angle is also matched to the Bragg reflection to be probed, allowing resolution to be optimized. The instrument has a sample table with 50 kg capacity and allows ≥170 mm translation along all three axes. A 1D position sensitive detector increases the instrument throughput significantly. The instrument was designed to have extremely low background levels so that sensitivity can be quite high. Unique beam aperture designs allow the gauge volume to vary from 1 mm^3^ to 125 mm^3^. DARTS is the most versatile high-resolution instrument in the United States for residual stress and texture measurements.

#### 5.2.4 Validation of Computational Methods

A Society of Automotive Engineers study of an induction-hardened steel shaft required determination of sub-surface residual stress as a function of fatigue. This proved to be a very interesting and difficult project because the composition of the steel varied from martensite at the surface to pearlite/ferrite in the center. This necessitated extraction of samples as a function of depth, after the main measurement sequence, to determine the “unstressed d-spacings” needed to obtain stress from strain [88,89]. One of the interesting aspects of this study is that finite element modeling (FEM) calculations had been made for the same system [90]. In general, validation of FEM calculations of residual stress by neutron diffraction represents a unique, long-term, significant contribution that the technique can make to technology. Another key area where validation of calculations is being actively pursued is for weldments, where subsurface stresses are very significant but inaccessible by other techniques [91,92].

#### 5.2.5 Residual Stress Standards Materials for Field-Portable Techniques

One of the very important uses of neutron diffraction residual stress measurement is in the characterization of samples suitable as standards and test specimens for field-portable techniques. This began in the 1980s with an aluminum ring/plug fabricated as a calibration sample for ultrasonics [85], continued with a steel ring/plug [92], and most recently includes another aluminum ring/plug specifically designed for testing an electromagnetic acoustic transducer (EMAT) system developed at NIST (Boulder) [93]. Other projects within the context of texture measurement included a study of magnetically aligned YBa_2_Cu_3_O_7_-type materials [94] and a still-continuing program aimed at developing methods by which reliable Rietveld refinements can be performed for textured materials [80,95].

#### 5.2.6 Recent Developments

The residual stress and texture program continues to find new applications. A relatively new and quite difficult application of neutron diffraction residual stress determination is in the area of coatings. Collaboration with the State University of New York at Stony Brook has produced some very interesting new results on a wide range of thick coatings [96,97]. Additional neutron intensity will allow the investigation of coatings and films in the less-than 500 µm thickness range. Improvements with this goal in mind are underway.

**Fig. 18:**
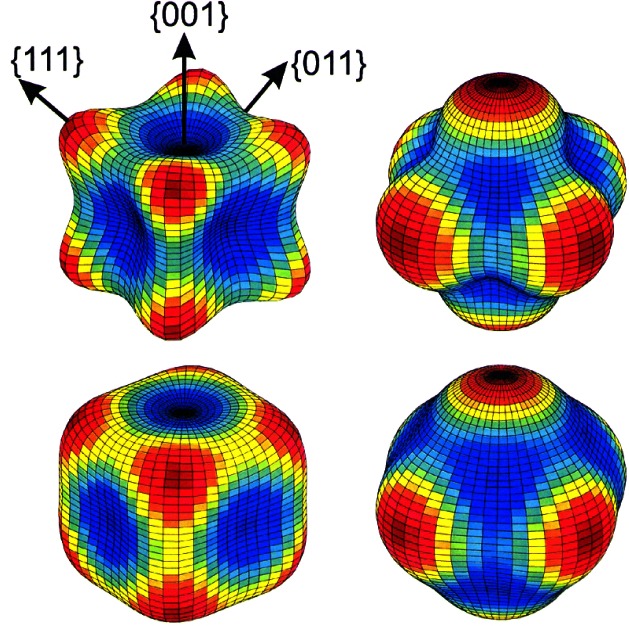
The upper and lower left figures, respectively, show the dependence of Young’s modulus on direction in single crystal cubic Ni and in polycrystalline Ni for a selection of crystallite grains having certain [*h,k,l*] lattice vectors that are parallel. The upper and lower figures on the right show a similar comparison for Poisson’s ratio.

Other recent work has been to develop a theory to allow determination of the fundamental single-crystal elastic constants of material from polycrystalline samples. This requires a theory to find the relationship between the elastic crystal lattice deformations of isolated grains in a polycrystalline matrix, i.e., lattice strain, to the applied macroscopic stress. Initial work done at the NCNR, as well as a separate effort at the Carnegie Institute in Washington, DC, has demonstrated solutions for single phase materials with high crystal symmetries and without preferred orientation [98,99]. Most recently, this technique has been extended for use with arbitrary polycrystalline aggregates of lower symmetry even in the presence of preferred orientation [100]. Some recent results are shown in Fig. 18.

## 6. Compositional Analysis of Materials by Nuclear Methods

### 6.1 The Need for Compositional Measurements

The use of nuclear methods at the NIST Center for Neutron Research provides elemental analysis with high accuracy, specificity, and sensitivity. They, along with other methods of analytical chemistry, contribute to a broad array of fields [101], since knowledge of the composition of materials is basic to their intelligent use. This is because chemical composition constrains atomic and macroscopic structure that affects many physical and engineering properties of materials such as mechanical, electronic, and transport behaviors.

### 6.2 Principles of Nuclear Methods of Analysis

Nuclear analytical methods generally depend on the occurrence of a nuclear reaction, resulting in either prompt or delayed radiation. Consider as an example a reaction commonly exploited in neutron activation analysis:

$${\;}^{23}\text{Na}{\left(\text{n,γ}\right)}^{24}\text{Na}\to^{24}\text{Mg}+{\text{β}}^{-}.$$

**Fig. 19:**
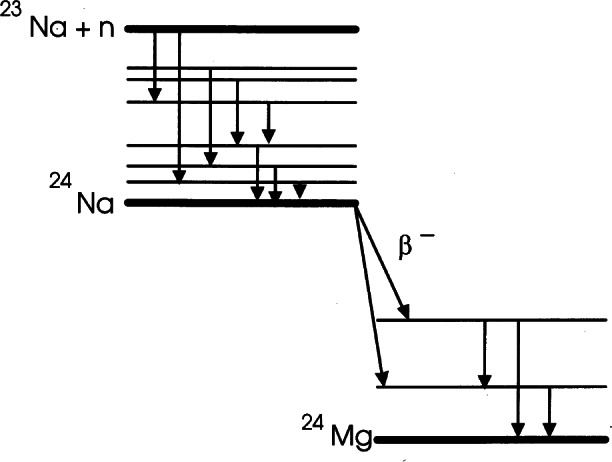
Schematic energy level representation of a simple neutron capture reaction.

Here a nucleus of stable sodium-23 absorbs a low-energy neutron. (See Fig. 19) Because ^24^Na has less mass (by 6.96 MeV/c^2^) than does the sum of ^23^Na plus a neutron, the neutron’s binding energy is emitted in less than 10^−15^ s as a cascade of over twenty gamma rays with characteristic energies. By collecting these gammas with a germanium detector during the neutron irradiation, the resulting spectrum may be used for identification and quantitative determination of sodium by prompt-gamma activation analysis (PGAA). After the capture takes place, the resulting ^24^Na nucleus in its ground state is more massive (by 5.52 MeV/c^2^) than the sum of a ^24^Mg nucleus plus a negative beta particle. It then decays with a half-life of 15 h, emitting a beta particle, a neutrino, and another group of characteristic gamma rays. In conventional neutron activation analysis (NAA), a Ge detector is used to measure these gamma rays for qualitative and quantitative analysis. These two methods are complementary: most of the elements in the periodic table can be determined by PGAA, NAA, or both.

Some special nuclides (notably ^10^B, ^6^Li, ^10^B, and ^14^N) emit energetic protons or alpha particles upon absorbing a slow neutron. The charged particle has a definite initial energy, which decreases by ionization at a known rate as it passes through matter. If the energy spectrum of these charged particles is measured, the depth distribution of the emitting nuclide is measured by this method, called neutron depth profiling (NDP).

Quantitation in NAA, PGAA, and NDP is most accurately performed by irradiating comparators of known quantities of the elements of interest [102,103]. An essential feature of these methods of analysis is that nuclear reactions are not influenced by the chemical state of the analyte. Most nuclear methods are purely instrumental, with no need to dissolve the sample before the determination step, a crucial operation that can lead to large errors in the analysis of complex materials [104]. In the certification of Standard Reference Materials (SRMs), this independence of chemistry provides a stringent cross-check of other methods [105].

### 6.3 Neutron-Based Analysis at NIST

The Radiochemical Analysis Section at NBS was established in 1963, even before the NBSR became available. The NIST reactor has a number of favorable characteristics for NAA: several large 40 mL pneumatic “rabbits” for sample irradiation, a high and well-thermalized neutron flux, and a long operating fuel cycle at stable power. For special purposes, irradiations can be performed in the thermal column (in which the ratio of thermal to fast neutrons is 1000) or in the reactor core (at a neutron flux of 4 × 10^14^ cm^−2^ s^−1^).

High-accuracy elemental analysis has been pursued at NBS since its beginning, especially for the characterization of SRMs. Systematic research in nuclear methods has reduced the uncertainty of all analytical steps, notably by characterizing the reactor neutron field [106], and reducing rate-related nonlinearities in counting [107]. As a result, the Analytical Chemistry laboratory at the NCNR has performed some of the most accurate activation analyses reported in the literature. To calibrate standards for Rutherford backscattering analysis, the masses of evaporated gold films on silicon were measured by NAA and gravimetry with agreement to 0.3 % [108]. By controlling errors from count-rate effects and peak integration methods, gamma spectrometry was used to measure the isotopic composition of uranium SRMs for nuclear safeguards, agreeing with UF_6_ gas mass spectrometry to better than 0.1 % [109]. More recently, NAA has been used as a primary ratio method to certify an SRM for the amount of arsenic implanted in silicon (useful to the electronics materials industry) with a relative standard uncertainty of 0.2 % [110].

The use of neutron beams for chemical analysis was pioneered at the NBSR. A prompt-gamma activation analysis system was installed by the University of Maryland in 1978 [111,112] and has been in continuous use ever since by scientists from the University, the Food and Drug Administration, and NIST. Along with two comparable installations built at about the same time [113, 114], this system inaugurated the modern combination of high-power neutron beams and large germanium gamma spectrometers for sensitive multi-element PGAA. More than 12 000 capture-gamma spectra have been accumulated at NIST in the analysis of a wide variety of materials.

When the Cold Neutron Research Facility became operational in late 1990, the first measurement made in the guide hall was by PGAA. NIST established the world’s first permanent cold-neutron PGAA spectrometer [115,116] and still has the highest-flux facility in service anywhere [117]. Planned revisions to the sample environment should improve its sensitivity and versatility still more.

Neutron depth profiling (NDP) has been developed into a mature analytical method at NIST [118]. A thermal-neutron instrument proved the usefulness of NDP in studying depth distributions of helium, lithium, boron, and nitrogen in a variety of scientific and technological applications. The current cold-neutron instrument has the highest flux in the world.

### 6.4 Applications to Materials Science

Neutron activation analysis has contributed to the characterization of a range of materials, from semiconductor silicon [119,120] to steel [121]. A review of neutron beam analysis at NIST [122] has 71 references to applications and also to methods. In fact, continual interaction between compositional analysis and neutron scattering has enriched both fields.

An especially important element is hydrogen, which can be measured by PGAA [123] but by few chemical methods. For example, nondestructive measurement of high levels of H in a titanium-alloy turbine blade indicated a possible reason for failure [124]. This work has led to the production of new Standard Reference Materials and to calibration standards for neutron tomography. Since hydrogen has a very large incoherent neutron scattering cross-section, a small amount of H contaminant can lead to measurement problems in neutron scattering studies. Consequently, hydrogen and other elemental concentration measurements by PGAA are regularly performed to complement neutron scattering at the NCNR [125, 126, 127].

### 6.5 Present Status and Near Future of Nuclear Methods at NIST

NIST has provided leadership in the development and application of nuclear methods of analysis by performing the only complete analysis of sources of error in NAA, which is required to demonstrate a primary method of analysis. Neutron fluxes and availability of the PGAA and NDP instruments at the NCNR facility are currently unmatched. This leadership was recognized recently as NIST hosted the Tenth International Conference on Modern Trends in Activation Analysis in1999 which was attended by over 200 scientists from 37 different countries.

In the near future, several elements with short-lived activation products will become measurable by NAA with the full commissioning of a rapid rabbit system, which transfers a sample from the reactor to the detector in less than 500 ms [128, 129]. Experiments are underway to use a chopped neutron beam for PGAA to discriminate against or in favor of capture products with lifetimes in the millisecond range [130]. Narrow, intense focused neutron beams from capillary optical lenses [131] are being used for spatially resolved PGAA [132] and NDP [133]. Plans are being made to relax the confined geometry for the cold PGAA beam, which will dramatically improve the hydrogen and fast-neutron backgrounds and permit the analysis of large samples. Neutron incoherent scattering is a promising new method [134] for hydrogen measurement, with better sensitivity (but less specificity) than PGAA. With currently available high quality capabilities and these planned developments, nuclear analytical chemistry methods at the NCNR can be expected to serve the NIST mission in materials research well into the future.

## 7. Chemical Physics of Materials

### 7.1 Chemical Dynamics Studied With Neutrons

Because of the neutron’s unique properties such as its unusually large scattering cross section for hydrogen, it was evident by the early 1960s that thermal and cold neutrons were destined to be invaluable probes of condensed-phase materials, particularly those containing hydrogen. For more than three decades, the large and diverse body of important chemical physics results obtained for hydrogenous as well as non-hydrogenous materials at the NCNR has been a testament to the unique power of neutron scattering methods for unraveling the structural and dynamical nature of a wide variety of condensed-phase materials. Below, we present a flavor of the chemical physics of materials research undertaken at the NCNR in order to provide some sense of the historic, current, and future aspects of these studies.

### 7.2 Hydrogen-Metal Systems

Hydrogen-metal interactions underlie a variety of critical technological issues such as hydrogen embrittlement, hydrogen storage, nuclear fusion, batteries, heterogeneous catalysis, and future energy production. NIST recognized early the importance of neutron scattering for elucidating the nature of these interactions as evidenced in part by the bestowal of NIST’s 1993 Samuel Wesley Stratton Award to J. Michael Rowe and John J. Rush for their pioneering studies of the submicroscopic behavior of hydrogen isotopes in metals. The NCNR has maintained a leadership role in this area with an active research program.

A large fraction of this research has historically involved the characterization of the site potentials for hydrogen absorbed in metal lattices and adsorbed on metal surfaces using neutron vibrational spectroscopy. The first measurements of the vibrational spectra for yttrium and uranium hydrides and deuterides [135] using the energy-gain scattering of cold neutrons (at Argonne National Laboratory) represented the beginning of hydrogen-metals research at the NCNR. The first measurements of the dynamics of metals containing hydrogen or deuterium by coherent neutron scattering were for the acoustic modes of PdH_0.03_ [136] followed by the detailed mapping of the phonon dispersion curves for PD_0.63_ by [137] and CeD_2.12_ [138] along the major symmetry axes for both the acoustic and optic modes. These seminal single-crystal measurements in conjunction with theoretical models helped unravel the relationship between the unusual lattice dynamics and the array of interatomic potentials associated with the different metal-hydrogen systems.

**Fig. 20:**
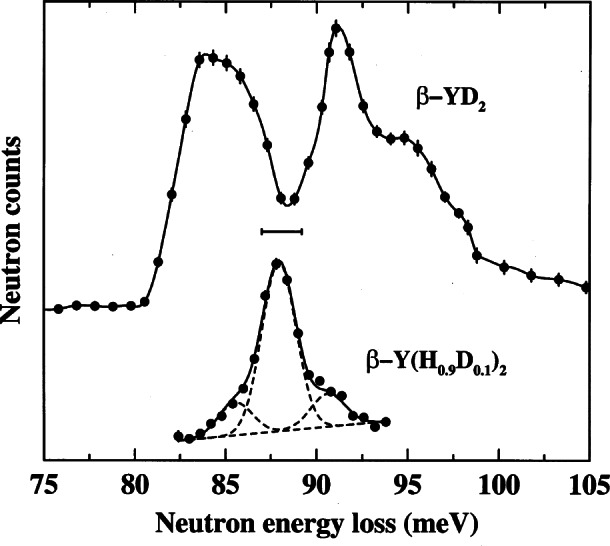
High-resolution deuterium (D) vibrational spectra for YD_2_ and Y(H_0.9_D_0.1_)_2_ below 10 K. A multicomponent Gaussian fit of the latter shows a strong central peak associated with the vibrations of isotopically isolated D atoms and a weaker doublet assigned to the localized acoustic and optic vibrations of isolated D-D pairs.

The construction of the BT-4 filter-analyzer neutron spectrometer with its superior signal/noise ratio led to the routine characterization of hydrogen vibrational signatures for virtually every class of hydrogen-metal system and spawned a variety of noteworthy spectroscopic studies by the early 1980s. These included such topics as hydrogen adsorbate dynamics on metal surfaces, unusual hydrogen ordering phenomena in the rare-earth metals, hydrogen tunneling in refractory metals, and the effects of isotope dilution on the vibrational dynamics of H and D in and on various metals. Since the first vibrational measurements of dilute H in β-phase palladium deuteride [139], isotope dilution neutron spectroscopy has developed into a particularly powerful technique for elucidating the nature of hydrogen-hydrogen interactions in materials. This is exemplified by a comparison of the D vibrational spectra [140] for YD_2_ and Y(H_0.9_D_0.1_)_2_ in Fig. 20. Yttrium dideuteride possesses the CaF_2_ structure with deuterium atoms occupying all of the interstitial tetrahedral sites of the fcc metal lattice. Since these sites have cubic symmetry, the expected normal-mode energies should be triply degenerate. Yet the measured YD_2_ spectrum clearly indicates a complex bimodal structure, reflective of significant D-D dynamic interactions causing a dispersion-broadened optic band. This explanation is corroborated by the contrastingly sharp, triply-degenerate D optic band for Y(H_0.9_D_0.1_)_2_. In this case, the occurrence of dynamic coupling interactions between neighboring D atoms is minimized by isotopic dilution of the D atoms with hydrogen. Closer scrutiny of the sharp band due to isotopically isolated D atoms reveals an additional minor doublet component indicative of localized acoustic and optic branches of a small fraction of dynamically-coupled, isolated D-D pairs. The 5 meV splitting implies a D-D/Y-D interaction force-constant ratio of ≈6 %.

Besides vibrational studies, the NCNR has also had a longstanding interest in the diffusive and tunneling properties of hydrogen in metals via quasi-elastic neutron scattering (QENS) measurements using triple-axis, time-of-flight, and backscattering instrumentation. One notable study involved the characterization of fast localized hopping of hydrogen atoms absorbed in the tetrahedral interstices of rare-earth metals [141]. In particular, a striking increase was measured in H hopping rates between near-neighbor H sites along the c direction in α-ScH*_x_* with decreasing temperature below 100 K (reaching 10^12^ s^−1^ at 10 K). The approximate *T*^−1^ dependence observed for the quasi-elastic linewidth pointed to nonadiabatic behavior associated with weak coupling of a quantum two-state system to the conduction electrons, the first such observation in a pure hydrogen-metal system.

### 7.3 Molecular Systems

Condensed-phase molecular systems are an intriguing class of materials in which the underlying lattice is comprised of molecules rather than individual atoms. The NCNR investigations of molecular systems started in the mid 1960s and focused on the spectroscopy and dynamics of large-amplitude intramolecular motions, notably (optically inactive) torsional vibrations and hydrogen-bond modes. For example, a neutron vibrational spectroscopic study [142] of the torsional mode in a prototype single-methyl-top molecule 1,1,1-trichloroethane (CH_3_CCl_3_) pinpointed the methyl torsional vibration at (37±2) meV, resulting in a calculated rotational energy barrier of (24±2) kJ/mol, which is almost double the previous value determined by thermodynamic measurements and infrared combination bands. This experimental finding was key to understanding the origin of rotational barriers in substituted ethanes. Later studies of solid nitromethane (CH_3_NO_2_) nicely illustrated the unique capability of low-energy neutron spectroscopy to reveal non-bonded molecular potentials in condensed-phase systems [143,144]. Measured methyl torsional energy levels for both CH_3_NO_2_ and CD_3_NO_2_ showed that the rotational potential was more complicated than a simple threefold or sixfold cosine potential. Moreover, increasing the pressure was found to increase the ground-state rotational tunnel splittings and decrease the torsional energy levels, a behavior contrary to previous studies of hindered rotors in the solid state. This anomalous pressure dependence was successfully explained by assuming a simple Lennard-Jones pairwise potential acting between atoms in the molecules.

In the 1970s and 1980s, substantial interest developed in characterizing the translation-rotation coupling in ionic and molecular solids. This coupling is the main determinant for such phenomena as incommensurate structures involving molecular orientations, phase transitions in orientationally disordered solids, and plastic and liquid crystal behavior. Examples of noteworthy NCNR work include the studies of the orientational properties of NH_4_^+^ ions in ammonium halides [145,146] and CN^−^ ions in alkali cyanides [147,148].

#### 7.3.1 The Fullerenes and Cubane

More recently, studies have been undertaken to elucidate the dynamical properties and their relation to structure of carbonaceous molecular solids such as the nearly spherical buckminsterfullerene (C_60_) [149,150], and cubane (C_8_H_8_) in which the carbon atoms reside at the corners of a cube with the H atoms outside of them along the body diagonals [151,152]. C_60_ displays classic plastic crystal behavior. It has a fcc phase above 260 K in which the molecules are tumbling nearly randomly, as was demonstrated by coherent QENS and diffraction studies on a powder sample. A simple rotational diffusion model described the energy and *Q* dependences and the diffuse diffraction pattern very well. Below this transition temperature the molecules were shown to liberate around particular orientations in a simple cubic $Pa\bar{3}\text{phase}$. Subsequent studies have focused on C_60_ compounds, C_70_, and nanotubes.

**Fig. 21:**
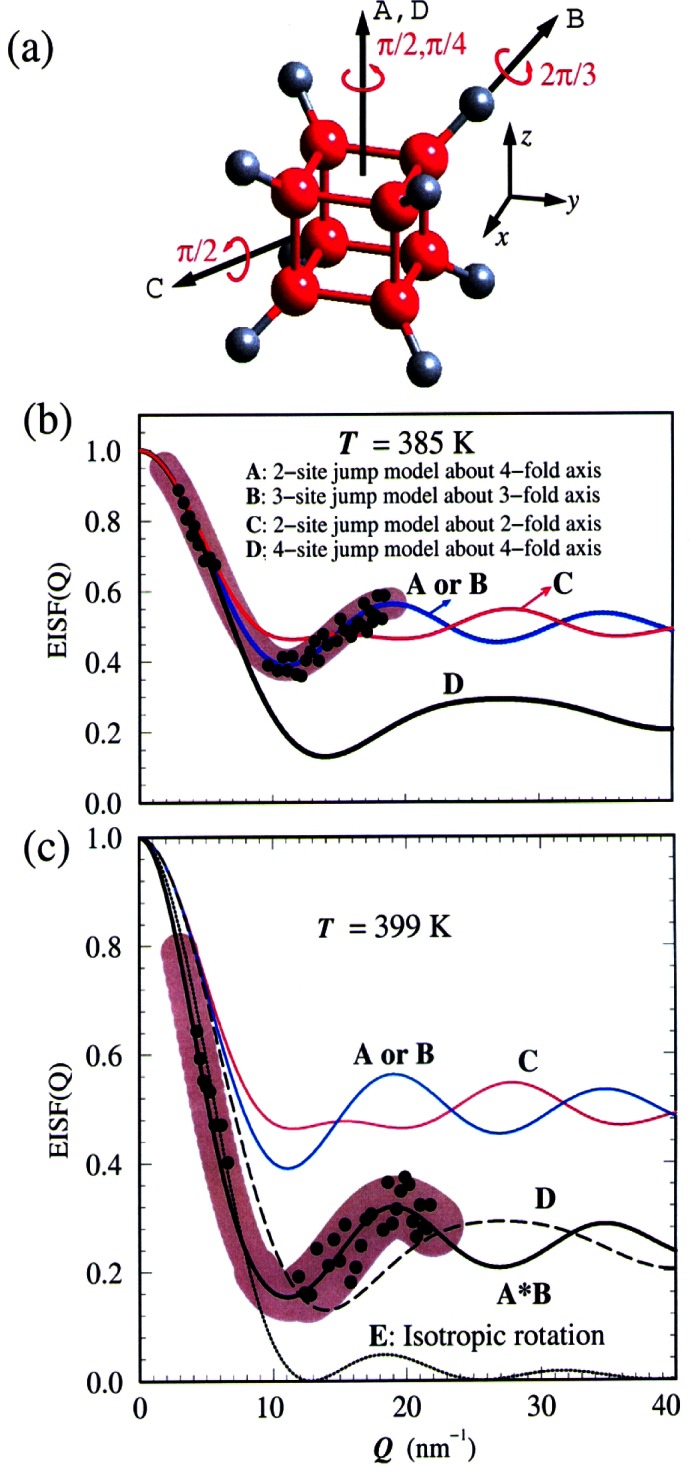
(a) Cubane molecule in its standard orientation. Various jump models about its principle axes are briefly described in (b) and also labeled (A,B …) on the molecule. (b) Elastic incoherent structure factors (EISF) for various jump models (solid lines) and experimental data (circles) in the orientationally ordered phase at *T* = 385 K. The broad line is a guide to the eye and indicates an upper bound on the uncertainties in the EISF vs *Q*. (c) Same as (b) but now the experimental data are for *T* = 399 K (in the orientationally disordered phase.) The data are fitted best by a model (labeled by A*B) involving A and B types of dynamics simultaneously.

Cubane’s unique geometry imposes an angle of 90° on the C–C–C instead of the 109.5° normally found in *sp*^3^ bonding, resulting in enormous strain energy. Solid cubane has been found to undergo a transition at 394 K from a low-temperature, orientationally ordered phase $\left(R\bar{3}\right)$ to a high-temperature, noncubic, orientationally disordered phase ( $R\bar{3}$ or *R*3*m*). Time-of-flight (TOF) spectroscopy has been particularly useful for characterizing both the vibrational density of states and the reorientational dynamics associated with solid cubane. The molecular librational modes are found to soften as the temperature increases, eventually overlapping with the molecular translational modes, leading to the 394 K order-disorder transition. Figure 21 illustrates the reorientational dynamics data along with various models based on jump rotations about the principle axes of cubane. In the ordered phase (at 386 K) close to the orientational phase transition, the elastic incoherent structure factor (EISF) is consistent with both π jumps about the fourfold axis and 2π/3 jumps about the threefold axis of the cubane molecule. Model calculations predict a much smaller barrier for the former reorientation geometry, making it the favored mechanism. The reason for the inconsistency with π/2 jumps about the fourfold axes is not yet clear, but this type of behavior is also seen in other molecular systems and must have something to do with the cooperative dynamics of the molecules. In the disordered phase (at 400 K), unlike the plastic phases of most molecular solids, the EISF results exclude single rotational-jump models as well as isotropic and free uniaxial rotational diffusion models. The best agreement is obtained by assuming an uncorrelated combination of π jumps about the fourfold axis and 2π/3 jumps about the threefold axis.

### 7.4 Heterogeneous Systems

Although not part of the traditional focus of the Chemical Physics of Materials program at the NCNR, momentum has grown over the years toward the application of neutron scattering methods to a variety of more complex real-world materials that are of significant fundamental and/or technological interest and that can be loosely classified as heterogeneous or guest-host systems. In these types of systems, a host lattice (usually porous) provides the stabilizing environment for the containment of guest molecules, typically hydrogenous in nature. This guest-host arrangement describes the morphology of an array of systems studied at the NCNR, including guest molecules such as hydrogen, water, and organic moeities absorbed or encaged within host materials such as zeolites, fullerenes, pillared clays, porous glasses, polymer membranes, and biological macromolecules. The nanoscaled confinement of the guest molecules and the particular nature of the guest-host interactions often leads to unusual structural and dynamical properties.

**Fig. 22:**
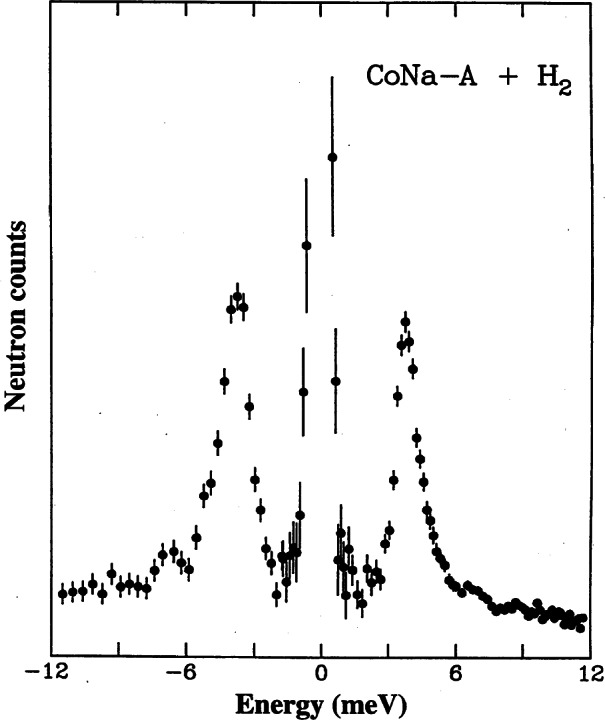
Time-of-flight (TOF) spectrum of 0.5 adsorbed H_2_ molecules per supercage in Co_4.1_Na_3.8_–A zeolite at 12 K. The spectrum of the unhydrogenated zeolite has been subtracted from the data. Positive energy represents neutron energy loss.

Figure 22 illustrates a TOF spectrum from a rotational-tunneling study of molecular hydrogen adsorbed in the cavities of partially cobalt-exchanged type-A zeolite. The assignment of the 3.8 meV neutron-energy-gain and energy-loss features to the rotational ground-state tunnel splitting of H_2_ [i.e., between *J* = 0 (para H_2_) and *J* = 1 (ortho H_2_) rotational states] was unambiguous since the expected intensity ratio between gain and loss processes would have been 1:40 at 12 K for a translational excitation. This assignment was in good agreement with a model for the H_2_ molecules in a twofold cosine potential with two degrees of rotational freedom. The model implied that the H_2_ molecules are bound end-on to the cobalt cations and perform 180° reorientations with a barrier of 55 meV to 68 meV.

#### 7.4.1 Studies of Hydration *in situ*: Cement

**Fig. 23:**
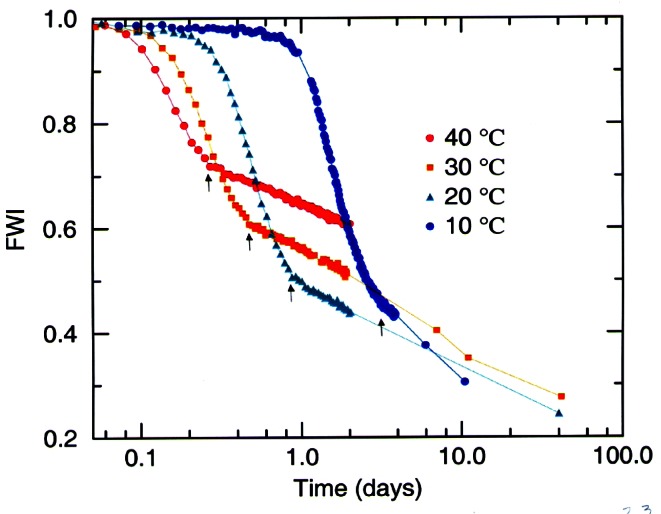
The free water index (FWI) vs time for Ca_3_SiO_5_ samples cured at different temperatures. The arrows indicate the sharp change in slope of the FWI, at which point the reaction changes from a nucleation and growth to a diffusion-limited behavior.

Recently, in conjunction with the Federal Highway Administration, QENS was used to study the hydration mechanism for tricalcium silicate (Ca_3_SiO_5_) [154]. The reaction between Ca_3_SiO_5_ and water is the principal factor in the setting and hardening of Portland cement, a heterogeneous system with obvious vast infrastructure applications. The kinetics of the reaction and even the specific mechanism are still not completely understood. The overall hydration process, which proceeds in several reaction steps, can be summarized as Ca_3_SiO_5_+(3+y−x)H_2_O→(CaO)*_x_* (SiO_2_)·(H_2_O)*_y_*+(3−*x*) Ca(OH)_2_. At any given reaction time, the TOF spectra reflected, via the accompanying quasi-elastic broadening, the fraction of water molecules which were still free and liquid-like (i.e., the free water index, FWI). As was expected, the FWI decreased throughout the hydration process as the free water was consumed by reaction with the Ca_3_SiO_5_. The *in situ* data taken at a series of fixed temperatures (see Fig. 23) showed that the FWI in the system remains relatively constant during an initial temperature-dependent induction period. This is followed by an exponential decrease due to nucleation and growth of the (CaO)*_x_* (SiO_2_)·(H_2_O)*_y_* gel over the surface of the exposed Ca_3_SiO_5_ grains. Finally at later times, the reaction becomes limited by the rate at which water can diffuse through the gel surface layer to reach the unreacted gel-encapsulated Ca_3_SiO_5_ grains. This novel experimental approach provided a much more direct measure of free-to-bound-water conversion as well as insights into the mechanism than other previously used methods.

#### 7.4.2 Protonic Conduction

Current work [155] in conjunction with DuPont on the dynamics of water in Nafion® (DuPont registered trademark polymer),^5^ a perfluorosulfonic acid ionomer, at the NCNR has been prompted by its use as a separator membrane in a variety of electrochemical applications such as electrolysis, fuel cells, and batteries. However, the relationship between Nafion’s useful macroscopic properties and its unusual microstructures is still not firmly established. A clear understanding of the ionic conductivity properties of hydrated Nafion membranes requires a detailed physical and chemical understanding of the water-Nafion interaction. Characterization of such a system illustrates the synergism among the different neutron scattering techniques used to probe materials properties. For example, neutron prompt-gamma-ray activation analysis is used to verify the water content for the various hydrated Nafions measured. Neutron diffraction and SANS are used to characterize the ionomer ordering and micellar-like clustering morphology of water molecules within the ionomer matrix. Neutron vibrational spectroscopy is used to characterize the hydrogen-bonding interactions of the guest water molecules intercalated within the host ionomer. Finally, quasi-elastic neutron scattering is used to characterize the water dynamics. The combined results from these neutron techniques allows for a more thorough understanding of the factors responsible for the favorable protonic conductivity properties associated with this material. So far, preliminary QENS spectra for dry and hydrated Nafions indicate that the superacidic protons originally associated with the sulfonic-acid (−SO_3_H) sidegroups are delocalized by the addition of water molecules to form H^+^(H_2_O)*_n_* clusters, a result in agreement with vibrational spectroscopic data. It is clear that the ability of water to abstract the superacidic protons from the sulfonic acid groups is intimately related to the unusual morphological and favorable conductivity properties associated with this hydrated ionomer. The quasi-elastic broadening associated with the clusters is indicative of a dynamical behavior akin to the restricted diffusion of water molecules within a nanosized sphere. This behavior can be correlated with the SANS data, which has provided morphological information concerning the size, shape, and spatial arrangement of the clusters.

### 7.5 The Future

Since its beginnings almost four decades ago, the Chemical Physics of Materials Program at the NCNR has dramatically expanded in both experimental capabilities and the scope of materials investigated. With the development and continual improvement in computing technology, the future focus will be on the more routine integration of *ab initio* computational methods with experimental results in order to gain a more fundamental physical description, from first principles, of technologically important materials properties.

## 8. Polymers and Complex Fluids

### 8.1 Using Neutrons to Study Large Molecules, Aggregates, and Polymers

Neutron scattering techniques began to be applied to polymers and complex fluids, materials with self-assembled or chemically bonded large molecular aggregates, in the early 1970s in Europe where the first specialized instruments to measure small-angle scattering were built. SANS quickly became one of the most important tools for investigating polymer microstructure as it became recognized that the scattering from a polymer molecule could be enhanced relative to its surroundings by substituting deuterium (^2^H or D) for some or all of the hydrogen (^1^H) atoms in the molecule. This technique of isotope labeling led to the resolution of many long-standing questions about the configurations of polymer molecules and the ways in which they pack together. Experiments on molten homopolymers in the mid-1970s, incorporating a small fraction of deuterated polymer chains, confirmed, for example, the decades-old prediction by Flory that the individual chains would adopt random-walk configurations without any chain swelling due to excluded-volume effects.

### 8.2 Early History of Polymer Studies at the NCNR

Stimulated by the SANS results emerging from Europe, NIST scientists Charles Han and Bernard Mozer set up a rudimentary SANS instrument at a thermal beam port (BT-5) at the NBSR. The instrument was about 4 m long with a 1 m long, linear position-sensitive detector set back two meters from the sample position to collect the small-angle scattering. A slotted rotating drum served to transmit relatively low velocity (long wavelength) neutrons from the tail of the Maxwellian reactor spectrum onto the sample, which further facilitated access to the scattering corresponding to small scattering vectors. With this setup, scattering in the *Q*-range from about 0.2 nm to 1.0 nm^−1^, corresponding to structural features from roughly 6 nm to 30 nm, could be measured, albeit with long counting times required to separate typically weak signals from the rather high ambient background. Despite these limitations, Han, Mozer, and collaborators were soon making significant contributions to the growing body of data on polymeric materials that SANS was providing. Their data on chain deformation in stretched rubber, collected from samples with a small fraction of perdeuterated chains between crosslinks, for example, were the first to indicate that the junction points, and not the chain segments between the junctions, move in proportion with the macroscopic deformation produced by stretching (junction affine deformation) [156]. This again served to elucidate an important problem that had eluded other methods of investigation.

### 8.3 Advances in Macromolecular and Polymer Sciences

With funds obtained in 1978 through NBS’s first solicitation for “competence initiatives,” the Reactor Radiation Division constructed a more versatile, 8 m long SANS instrument with a large (64 cm by 64 cm) two-dimensional position-sensitive detector, thus enabling investigations of anisotropic SANS over a wider *Q*-range, 0.08 nm^−1^ to 5 nm^−1^ (sizes from 10 nm to about 80 nm). The availability of this instrument stimulated materials scientists throughout NBS and their outside collaborators to apply SANS measurements to characterize the microstructure of a wide range of materials. Applications in polymers and in solution studies of colloidal particles remained dominant, however, owing to the increasingly sophisticated use of H-to-D substitution techniques to control scattering contrast.

The burst of experimental activity in the application of SANS to polymers that took place in the late 1970s and early 1980s was driven in part by an equally strong and productive period of development in the theory of scattering from complex systems with isotopically labeled components. Here again NIST scientists made significant contributions. Han, in collaboration with A. Z. Akcasu, G. Summerfield, and others, showed in 1980 [157] that contrary to intuition and practice up to that time, it was not necessary to extrapolate results obtained on systems with labeled (i.e., deuterated) components to the limit of infinite dilution to determine the conformation of individual polymer chains. The-gradual recognition that followed that high concentration labeling gave results that were not only interpretable, but more sensitive and precise,^6^ led to the successful study of many, often quite subtle, aspects of polymer phase behavior that would have been impossible if attempted in the dilute limit.

General expressions were developed by H. Benoit and W. Wu of NIST for the scattering from multicomponent systems such as mixtures of homopolymers and copolymers [158]. These provided the basis for interpreting scattering from, for example, block copolymers in the bulk, in mixtures with homopolymers, and in solutions, that in many cases were borne out in measurements carried out on the 8 m SANS instrument in the early to mid-1980s. In another important theoretical advance of this period, N. Berk of NIST developed analytic expressions for the scattering from a model bicontinuous structure [159] that remarkably accounted for previously unexplained features in the small-angle scattering from bicontinuous (Winsor III phase) microemulsions and mesoporous glasses (e.g., Vycor). Based on an algorithm originally invented by J. Cahn of NIST [160] to simulate morphology resulting from spinodal decomposition in metal alloys, the Berk model, as it is now widely known, provided the first unified description of scattering from bicontinuous microemulsions. Among its successes, Berk’s model accounted for the puzzling disappearance of the characteristic low-angle peak associated with the fundamental correlation length between the water and oil regions when the volume fractions and the (neutron) refractive indexes of these two major phases are matched.

#### 8.3.1 The Advent of Cold Neutron Instruments: SANS

High resolution SANS measurements, utilizing cold (long wavelength) neutrons and pinhole collimation over long distances, became possible for the first time in the United States when the neutron guide hall and cold neutron source were installed at the NBSR in the late 1980s. The first new instrument to operate in the guide hall (in 1991) was a 30 m long SANS machine with a large area detector that moves along rails inside a 15 m long vacuum vessel to cover a *Q*-range from 0.015 nm^−1^ to 7 nm^−1^ corresponding to sizes up to about 400 nm. Built jointly with the Exxon Research & Engineering Company (now Exxon Mobil) and the University of Minnesota, this instrument was designed to be versatile and user friendly [162] in keeping with the NCNR’s mandate to operate as a national user facility. By the time this instrument was completed in 1991, demand for SANS measurement time was such that the National Science Foundation funded the construction and operation of a second 30 m SANS instrument that has been in operation in the guide hall since 1992.

The capabilities afforded by the 30 m SANS instruments made possible new areas of application to polymers and complex fluids. The higher intensity available under moderate resolution conditions made it possible to record complete SANS patterns in a few minutes. This capability was exploited by Han and coworkers [162] to make definitive measurements of the kinetics of spinodal decomposition in polymer blends and by Bates, et al. [163] to follow microphase separation in block copolymers, for example. In another first-of-its-kind experiment, Co and Kaler [164] succeeded in making time-resolved measurements of the growth kinetics of latex particles formed by polymerization of monomers solubilized in a microemulsion.

**Fig. 24:**
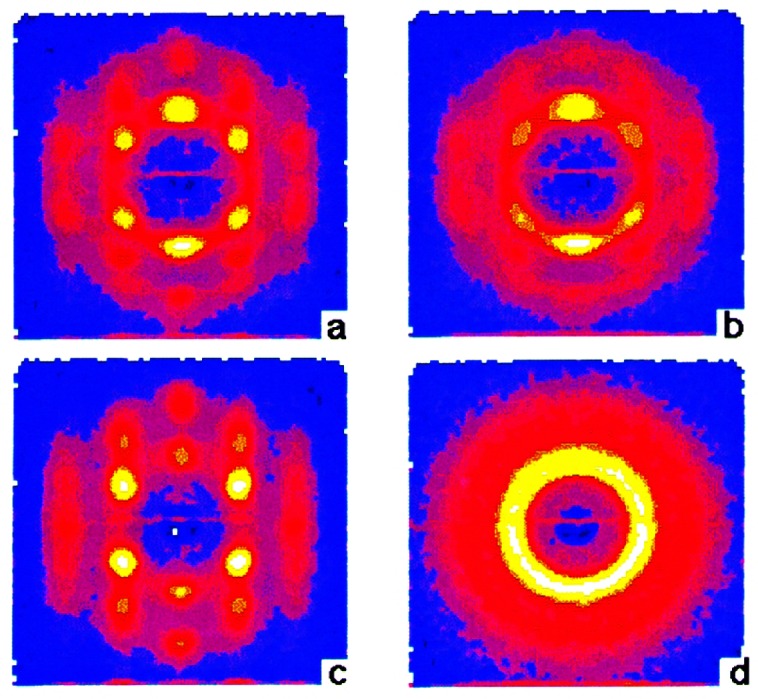
Block copolymer micelles under shear: color-coded images of the small angle neutron scattering (SANS) from a solution of block copolymer aggregates, micelles of polystyrene-polyisoprene in decane, as recorded on the two-dimensional detector at one of the NCNR’s 30 m SANS instruments. The measurements [165] were made in a flow cell at shear rates from zero (a), to 300 Hz (d), and show a progression from an ordered bcc structure to a disordered liquid-like structure at high shear.

Interest in recent years has shifted from structural studies under static ambient conditions to measurements in applied fields and non-equilibrium conditions related to materials processing environments. There have been several extensive studies of colloidal solutions under shear flow, for example, that have provided new insights into the shear-induced microstructural changes that result in shear thickening or shear thinning, depending on the dominant interactions involved. In one of the most complete shear flow studies done to date, Gast et al. [165] examined the self-assembled aggregates, analogous to surfactant micelles, that diblock copolymer molecules form in selective solvents, i.e., solvents in which one block is miscible (and hence swells) but the other is not (and hence collapses on itself). Depending on the concentration and the relative lengths of the two types of polymer in the molecule, the aggregates are driven by moderately long-range osmotic forces to form long range ordered crystal structures. Even more remarkably, the polycrystalline structures that form in equilibrium can be oriented by steady shear over macroscopic dimensions, as evidenced in the SANS patterns shown in Fig. 24. By systematically studying how these ordered structures evolve with concentration, relative block size, and shear rate, Gast et al. [165] were able to obtain detailed information on the underlying interaction potential pertaining to such systems.

Another thermodynamic parameter that influences polymer processing and properties is pressure. For example, Balsara et al. [165–169] have made effective use of pressure to map out spinodal and nucleation phase separation lines in polyolefin mixtures by SANS measurements. Pressure jump experiments monitored the kinetics of demixing in the two-phase regions. These workers have carried out the first systematic study of the nucleation and growth process in such mixtures. During the early stage of growth, concentration fluctuations lead to slowly evolving clusters that then act as nucleating centers for the second rapid growth stage of phase separation. A nucleation shift factor describing the slowing down of nucleation kinetics with decreasing pressure quench depth has been estimated from their results. This time-pressure equivalence is reminiscent of the time-temperature superposition principle observed in polymer rheology.

Supercritical fluids can also be loaded into polymer materials under pressure. An example of this is the work of Watkins et al. [170] in which compressed CO_2_ is loaded into polymer blends and diblock copolymers. SANS studies of these systems exhibit dramatic reductions of their lower critical solution transition (LCST) and lower disorder-to-order transition (LDOT) upon absorption of a few percent CO_2_ (i.e., increase of the mass fraction of CO_2_ in the polymer blend by a few percent.) For example, the LDOT of symmetric poly(deuterated styrene)-block-poly(n-butyl methacrylate) copolymers having total relative molecular masses of 78 000 and 32 000, respectively, are depressed by as much as 250 °C upon exposure to supercritical CO_2_ at modest fluid-phase densities.

**Fig. 25:**
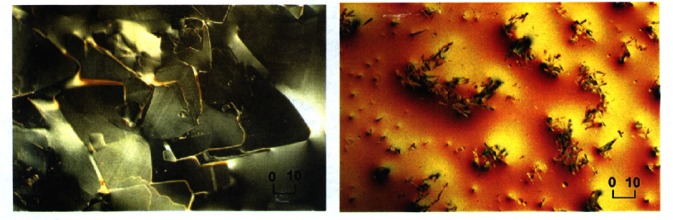
Optical micrographs of wax crystals that form in diesel fuel at −13 °C. The scale indicated in the lower right corner of the micrographs is in µm. The image on the left is for untreated diesel fuel in which large crystals grow that can clog fuel filters and cause engine stoppage. The image on the right is the same fuel treated with a type of diblock copolymer that scavenges the wax molecules thus preventing the growth of large crystals. The structure and function of the copolymer additive were determined by SANS measurements carried out at the NCNR [166] and other neutron research centers.

Basic SANS research carried out at the NCNR over the years on the phase behavior of polymers and complex fluids has not only markedly improved our understanding of such systems, but has led directly to industrial applications. ExxonMobil scientists, for example, have accumulated data on the relative miscibility of a wide variety of polyolefin blends that form the basis for processing new industrial plastics with improved properties. Basic studies of the solution properties of block copolymers, similar to those of Gast et al. mentioned above, led ExxonMobil scientists to infer that a particular class of copolymer would be effective in scavenging wax molecules from diesel fuel, thus inhibiting the formation of large wax crystals that could clog fuel lines at low temperatures (see Fig. 25). In less than 3 years from the time the initial SANS studies [171] (carried out at the NCNR and other neutron research centers in Europe) confirmed this behavior, an additive that keeps diesel fuel fluid at temperatures down to −30 °C was developed and successfully field-tested.

#### 8.3.2 The Advent of Cold Neutron Instruments: Reflectometry

The organization of macromolecules at surfaces and interfaces has enormous significance for industrial applications involving coatings, surfactants, inks, and membranes, and phenomena such as wetting, drag reduction, corrosion, and adhesion. Since SANS is not particularly sensitive to near surface structure, progress in these areas required the development of the specialized, surface-sensitive technique of neutron reflectometry. The phenomenon of reflection and refraction of neutrons by materials was first studied by Fermi and Zinn in 1944 at the University of Chicago. The neutron refractive index for most materials, however, is slightly less than one, but only by between (1×10^−6^ and 10×10^−6^). This gives rise to total external reflection of neutrons by most materials, although only at shallow glancing angles. Along with the facts that neutrons are a penetrating probe and have wavelengths that are comparable to interatomic distances, this has been exploited by researchers to study the structure of surfaces and interfaces in thin films and multilayers using the technique of neutron reflectivity.

The technique was introduced in Europe in the mid-1980s, and NIST played a leading role in its development almost from the beginning. In 1987 Charles Majkrzak and Sushil Satija started doing neutron reflectivity by converting the BT-4 spectrometer at NBSR to obtain very narrow ribbon like neutron beams required for these measurements. They quickly showed that measurements of reflectivity as small as 1×10^−6^ were possible with this type of setup. A first set of neutron reflectivity experiments was performed in collaboration with IBM scientists to study the self-assembly of thin films of diblock copolymers of polystyrene (PS) and polymethylmethacrylate (PMMA) into a multilayer morphology [172]. For neutron reflectivity studies of these multilayers, the PS blocks were deuterated and the PMMA blocks hydrogenated. These studies showed in exquisite detail the morphology of these films, for example, how sharp the interfaces between the PS and PMMA blocks are, the shape of these interfaces, and the interlayer spacing between PS and PMMA layers. Such details were not possible to obtain before and these new and highly important results have led to rapid growth of such studies in various aspects of polymer interdiffusion in thin films by many groups worldwide.

In 1989 a dedicated neutron reflectometer was built at the beam port BT-7 in the NBSR. This new instrument allowed neutron reflectivity as small as 1×10^−7^ to be determined and facilitated measurements on solid-liquid interfaces. Since neutrons can penetrate through large perfect crystals of silicon or quartz, it is possible to send a neutron beam through such a crystal, which can reflect off the crystal-liquid interface with the reflected beam traversing back through the silicon crystal. The feasibility of such experiments was demonstrated by experiments on diblock copolymers adsorbed from solution at the silicon-solution interface [173]. It is worth pointing out the unique capabilities of neutrons in this regard since x rays cannot probe solid-liquid interfaces due to their limited penetration through solids. Since the first study of a solid-liquid interface, several experiments to probe the conformation of physisorbed as well as chemically grafted polymer brushes have been carried out. (A polymer brush consists of chain-like molecules with one end tethered to a surface and the tails standing extended away from the surface.)

With the availability of a new cold neutron source and neutron guide hall a new instrument was installed on the NG-7 beam line in 1993. This new instrument was built jointly with IBM Almaden and the University of Minnesota. It had four times more neutron intensity than the BT-7 reflectometer and also allowed horizontal positioning of samples in order to study free liquid surfaces and interfaces. The NG-7 reflectometer was built as a user-friendly machine to allow researchers from other institutions easy access to reflectivity measurements. Further studies on equilibrium properties of tethered polymer chains under varying solvent conditions were carried out by Karim et al. [174], which for the first time showed that the *in situ* conformation of these polymer brushes agrees very well with numerical simulations and self-consistent field calculations for both the good and theta solvent cases. The horizontal sample capability of the NG-7 reflectometer has been exploited in a number of studies of Langmuir films of polymers and block copolymers on free liquid surfaces. For example, a recent study of Langmuir monolayers of diblock copolymers in theta solvent condition [175] has pointed out important differences between the theoretically predicted scaling laws for the tethered chains and experimental observation. This has prompted further interest in the theoretical models for polymer brushes.

The conformation and dynamics of polymer chains at interfaces are often quite different from those of the bulk polymer. For example, geometric constraints from the interfacial surface alter the configuration of the polymer chains, and the interaction energy between the polymer and the surface can provide additional enthalpic contributions to the interfacial polymer properties. In a number of studies with neutron reflection [176], it was demonstrated that polymer interdiffusion near a surface is substantially slowed down due to the interaction of polymer chain segments with the surface. A present focus is on studies of reflectivity of thin polymer films at high pressure in supercritical CO_2_ fluid, conformation of polymer brushes under shear solvent flow, and wetting properties of oils on water surfaces.

The widely recognized impact that the NCNR’s high resolution SANS and reflectometry instruments have had on research in polymers, complex fluids and other areas of materials science is due in part to their broad measurement range and high sensitivity, but is largely due to their accessibility to researchers throughout the United States and the world through the NCNR’s formal user program that went into effect in the early 1990s. The NCNR’s SANS instruments now serve over 200 visiting researchers annually, and the reflectivity instruments over 100, from universities, government and industrial laboratories. One measure of the impact that these facilities have had on research and development in polymers and complex fluids is the number of major awards for work that included neutron scattering measurements carried out at the NCNR. A list is given in Table 1. In addition, publications of work done at the NCNR in the areas of polymers and complex fluids now exceed 100 per year, or roughly 40 % of the total number of publications.

### 8.3 Ultra-high Resolution SANS

New instrumentation continues to be developed at the NCNR that has particular applicability to the characterization of structure, as well as dynamics, of polymers and complex fluids. In 2000, the NCNR brought on-line the world’s highest intensity neutron Bonse-Hart-type, perfect crystal diffractometer for ultra-high resolution SANS. Using large perfect Silicon (220) triple-bounce, channel-cut crystals to achieve a beam profile with a full-width at half-maximum of only 8 µrad, corresponding to scattering at a minimum *Q* of about 0.002 nm^−1^, this instrument enables structural features up to about 3 µm in size to be quantified. With the addition of this unsurpassed capability, the SANS instruments at the NCNR are now sensitive to microstructure over four orders of magnitude, from 1 nm to 10 000 nm. This measurement range is needed to fully investigate the hierarchical structures typical of nanocomposite and biomimetic materials that are becoming increasingly important in the development of new functional and structural materials. For example, to fully describe the microstructure of polymer-clay nanocomposites that are being developed for their improved mechanical properties at higher temperatures requires probing dimensions on the scale of the natural platelet spacing in clays (1 nm to 2 nm), the dimensions characteristic of the polymer chains (10 nm to100 nm), and the lateral dimensions of the clay platelets (1 µm to 10 µm). Complete characterization of such highly anisotropic microstructure is possible with the combination of SANS instruments now available at the NCNR.

The forces and interaction potentials that determine the structures found in polymers and complex fluids are manifested in the motions of the constituent macromolecules. These motions occur on times scales, from 10^−10^ s to 10^−7^ s, which are accessible with cold neutron spectroscopic techniques such as are available on the very recently commissioned neutron backscattering (see Sec. 3.6 for a biological example) and spin-echo spectrometers. Both of these techniques were pioneered in Europe but since 1999 are now available for the first time in the United States at the NCNR. In both of these cases, however, the NCNR’s instruments incorporate advances providing U.S. researchers with the leading measurement tools available anywhere for analyzing the motions associated with, for example, shape fluctuations of micelles, vesicles and microemulsions, or the motions of protein domains.

The combination of state-of-the-art instrumentation in SANS, reflectometry, and neutron spin-echo and backscattering spectroscopy, and an access policy that is open to all qualified researchers, has made the NCNR the most productive neutron research center in the United States for studying the structure and dynamics of soft condensed matter systems.

## 9. Summary

During the 31 years since the first operation of the NBSR, as instrumentation has evolved to exploit their distinctive properties, neutrons have become an increasingly important probe in materials spanning a wide range of disciplines from biology to mechanical engineering. Examples are given above to demonstrate the power and breadth of this probe in making measurements to answer key scientific questions and in revealing key properties of virtually all classes of materials. The NIST Center for Neutron Research has become the most heavily used and scientifically active facility in the United States in this enterprise. With its continuing development of new instruments, the NCNR is poised to provide a unique service well into the future to the scientific, engineering, and technical communities focused on discovering new materials and applications central to future commerce.

## Figures and Tables

**Table 1:** Winners of scientific prizes for work in polymers and complex fluids carried out in part on the SANS and reflectometry instruments at the NCNR

Name	Award
Charles Han	1984 Dillon Medal, Division of High Polymer Physics, American Physical Society,
	1999 High Polymer Physics Prize, American Physical Society
Alice Gast	1992 Allan P. Colburn Award
Frank Bates	1989 Dillon Medal, Division of High Polymer Physics, American Physical Society,
	1997 High Polymer Physics Prize, American Physical Society
Nitash Balsara	1997 Dillon Medal, Division of High Polymer Physics, American Physical Society
Eric Kaler	1998 Award for Colloid Chemistry, American Chemical Society
Spiros Anastasiades	1998 Dillon Medal, Division of High Polymer Physics, American Physical Society
Anne Mayes	1999 Dillon Medal, Division of High Polymer Physics, American Physical Society
Lewis Fetters	2000 High Polymer Physics Prize, American Physical Society

## Footnotes

1 Whereas x rays scatter identically from ^1^H and ^2^H because they have the same electronic structure, neutrons scatter very differently, in both amplitude and phase, through interaction with the nuclei. A measure of the ability of an isotope to scatter slow neutrons is its scattering length, *b*, a length of the order 10^−15^ m, i.e., of the size of a nucleus, but that varies markedly and non-systematically with isotopic species.

2 From the diffraction relation, *d* ≈ 2 π/*Q*, between the size scale probed *d* and the magnitude of the scattering vector, *Q* = (4π/λ)sin(*θ*/2), where λ is the wavelength of the probing radiation and *θ* is the scattering angle, measurements at small angles (or using longer wavelengths) are necessary to probe larger scale structural features in materials.

3 The average scattering length density in a small volume surrounding some point in a sample is defined as the sum of the neutron scattering lengths (see footnote 1) for all the nuclei within that volume, divided by that volume.

4 In an amorphous system, the momentum *ℏ****Q*** and energy *E* transferred to the sample when a neutron scatters from it, introduced in section 1.1, must satisfy *E* = *E*_exc_ and *ℏQ* = *ℏq* in order to create a quantum of excitation. The excitation energy *E*_exc_ is related to *q* by a dispersion relation, at least for small *q*, as discussed later.

5 Certain commercial equipment, instruments, or materials are identified in this paper to foster understanding. Such identification does not imply recommendation or endorsement by the National Institute of Standards and Technology, nor does it imply that the materials or equipment identified are necessarily the best available for the purpose.

6 The scattering from a labeled homopolymer is proportional to the product *φ*(1−*φ*) where *φ* is the fraction of deuterated chains and hence is maximal when 50 % of the chains are labeled.
